# Quality indicators for osteoarthritis pain management in the primary care setting

**DOI:** 10.1186/s12891-023-06637-x

**Published:** 2023-06-30

**Authors:** Elsie Rizk, Sharla Tajchman, Ezekiel Fink, Dipendra K. Aryal, Tomona Iso, Eleazar Flores, Anthony E. Brown, Sagar P. Chokshi, Shetal-Nicholas Desai, Ashvin K. Dewan, Sarah A. Kazzaz, Myriam Guevara, Sudha Nagaraj, Christopher P. Robben, Veronica Vittone, Joshua T. Swan

**Affiliations:** 1 Department of Pharmacy, Houston Methodist, Houston, TX USA; 2 Department of Surgery, Houston Methodist, TX Houston, USA; 3 Pfizer, Inc., New York, NY USA; 4 Department of Neurology, Houston Methodist, Houston, TX USA; 5 Houston Methodist Primary Care Group, Houston Methodist, Houston, TX USA; 6 Department of Neurosurgery, Houston Methodist, Houston, TX USA; 7 Department of Orthopedic Surgery, Houston Methodist, Houston, TX USA; 8 Houston Methodist Academic Medicine Associates – Rheumatology, Houston Methodist, Houston, TX USA; 9 Center for Outcomes Research, Houston Methodist, Houston, TX USA; 10 Department of Surgery, Houston Methodist Hospital, 6550 Fannin Street, SM1661, Houston, TX 77030 USA

**Keywords:** Osteoarthritis, Pain, Quality indicators, Primary care, Opioid stewardship

## Abstract

**Background:**

Development of valid and feasible quality indicators (QIs) is needed to track quality initiatives for osteoarthritis pain management in primary care settings.

**Methods:**

Literature search identified published guidelines that were reviewed for QI extraction. A panel of 14 experts was assembled, including primary care physicians, rheumatologists, orthopedic surgeons, pain specialists, and outcomes research pharmacists. A screening survey excluded QIs that cannot be reliably extracted from the electronic health record or that are irrelevant for osteoarthritis in primary care settings. A validity screening survey used a 9-point Likert scale to rate the validity of each QI based on predefined criteria. During expert panel discussions, stakeholders revised QI wording, added new QIs, and voted to include or exclude each QI. A priority survey used a 9-point Likert scale to prioritize the included QIs.

**Results:**

Literature search identified 520 references published from January 2015 to March 2021 and 4 additional guidelines from professional/governmental websites. The study included 41 guidelines. Extraction of 741 recommendations yielded 115 candidate QIs. Feasibility screening excluded 28 QIs. Validity screening and expert panel discussion excluded 73 QIs and added 1 QI. The final set of 15 prioritized QIs focused on pain management safety, education, weight-management, psychological wellbeing, optimizing first-line medications, referral, and imaging.

**Conclusion:**

This multi-disciplinary expert panel established consensus on QIs for osteoarthritis pain management in primary care settings by combining scientific evidence with expert opinion. The resulting list of 15 prioritized, valid, and feasible QIs can be used to track quality initiatives for osteoarthritis pain management.

**Supplementary Information:**

The online version contains supplementary material available at 10.1186/s12891-023-06637-x.

## Background

More than 32 million adults in the United States suffer from osteoarthritis (OA), accounting for $373 billion in annual direct medical costs [1–3]. Persistent pain caused by OA impairs patients’ ability to perform activities of daily living, and pain management is an integral component of maintaining a good quality of life for patients with OA [2, 4]. Despite guideline recommendations to avoid or minimize opioid use for OA, opioids are prescribed in 26% of outpatient encounters for OA [5]. Approximately 35% of hip or knee OA patients who are treated with opioids will fail opioid therapy, defined as the need for higher opioid doses, addition of non-opioid analgesics, surgery, or opioid misuse [6]. An estimated 5% of knee OA patients are chronic users of strong opioids, which generates $14 billion in societal costs driven by medical care, lost productivity, diversion, and criminal justice [7]. Most patients with OA initially seek pain management at primary care clinics. Since primary care physicians (PCPs) are common prescribers of opioids for OA [8], they need to take an active role in optimizing the safety and efficacy of pain regimens for OA to reduce reliance on opioids and combat the opioid crisis that currently plagues the United States [9, 10].

Numerous national and international guidelines provide an abundance of recommendations regarding evidence-based strategies to optimize pain management while minimizing the risk of adverse events from pain medications. However, primary care practice does not always correlate with guideline recommendations, potentially due to lack of incentives for PCPs, lack of PCP buy-in, lack of integration between researchers and PCPs, and low perceived prioritization of OA among PCPs and patients [11–14].

A potential solution to improve adherence to guideline recommendations is to develop a research program that is embedded in a health-system and established through adequate buy-in from PCPs and related physician specialists. The initial step for this program is to develop a set of quality indicators (QIs) that are deemed to be meaningful and relevant among stakeholders. The objective of this consensus project was to develop a set of prioritized, valid, and feasible QIs that can be used to track quality initiatives for OA pain management in the primary care setting.

## Methods

### Setting

The Houston Methodist health system consists of 148 PCPs that practice at 39 locations in the greater urban area of Houston, Texas, USA [15]. In 2018, our Opioid Stewardship Program at Houston Methodist successfully established consensus on QIs for opioid stewardship and pain management in the hospital and emergency department settings [16]. This study applied a similar pragmatic modification to the Research and Development Corporation/University of California Los Angeles (RAND/UCLA) method to establish consensus on valid and feasible QIs for OA pain management in the primary care setting [17, 18]. Consensus was established using a 5-step mixed-methods approach: (i) literature search, (ii) feasibility screen, (iii) face validity screen, (iv) expert panel discussions, and (v) priority ranking. The Houston Methodist Research Institute’s Institutional Review Board approved this study with a waiver of informed consent.

### Composition of expert panel

Our OA expert panel consisted of healthcare leaders (medical director of primary care, medical director of pain, and the chief medical information officer), clinicians, and health services researchers from the Opioid Stewardship Program. This 14-member multidisciplinary team included 5 PCPs, 3 pharmacist researchers, 2 rheumatologists, 2 orthopedic surgeons, and 2 pain specialists.

### Literature search

A literature search identified practice guidelines for OA management that were published from January 2015 to March 2021 and indexed in MEDLINE (accessed via PubMed) or SCOPUS (Supplementary Methods 1 section of Additional File 1). Our search strategy was limited to recently published practice guidelines to represent the period of the publicly acknowledged opioid crisis in the United States that was declared in 2016–2017 [9, 19, 20]. Additionally, federal agency websites and professional society websites were reviewed for relevant position statements and guidelines that were not otherwise published and indexed. Only guidelines that contained evidence-based recommendations for management of OA in adults and were associated with a professional organization or government were included. Guidelines that exclusively focused on diagnosis or surgical management of OA were excluded. All abstracts identified were independently screened by two investigators for inclusion; discrepancies were settled by a third investigator.

Using a standardized electronic data collection tool, investigators extracted evidence-based recommendations from each included reference. For each recommendation, investigators also extracted information on applicable joints, comorbidities, and strength of recommendation. To focus on pain management in the primary care setting, recommendations related to OA diagnosis, intraoperative management, postoperative recovery, and assessment of postoperative outcomes were not extracted. Recommendations were consolidated into brief, commonly worded proposed QIs and organized into 11 domains: topical medications, intraarticular injections, biologics, systemic medications, support devices, supplements, alternative therapies, education, behavior and psychosocial interventions, procedures, exercise, and other. Proposed QIs were advanced to feasibility and validity screening processes.

### Feasibility screen survey

Feasibility was evaluated using an electronic survey to score each proposed QI as not feasible (score = 0) or feasible/unsure (score = 1) based on 2 feasibility screening criteria that were modeled after antimicrobial stewardship and opioid stewardship consensus methodology [16, 21]: (i) Assuming healthcare documentation is compliant with hospital policy and expectations, this QI can be reliably extracted from structured data fields within the electronic health record (EHR) (current state or future state); (ii) This QI is relevant to management of OA pain in a primary care clinic (family medicine or internal medicine). Five experts scored the feasibility of each proposed QI as 0 or 1, and proposed QIs with a total score of 3 to 5 were considered feasible and were retained as candidate QIs.

### Face validity screen survey

Face validity was evaluated using an electronic survey to score each candidate QI using a 9-point Likert scale (with 1 indicating lowest validity and 9 indicating highest validity) based on 3 face validity criteria that were modeled after antimicrobial stewardship and opioid stewardship consensus methodology [16, 21, 22]: (i) This QI is associated with improved OA pain management in primary care clinics (family medicine or internal medicine); (ii) This QI is associated with improved quality of care and patient safety; and (iii) This QI can be influenced by electronic medical record enhancements. Survey scores were used to preliminarily categorize candidate QIs as appropriate (median score > 6, without disagreement), inappropriate (median score < 4, without disagreement), or uncertain (disagreement or median score 4–6). Disagreement was defined as ≥ 5 ratings of 1–3 with ≥ 5 ratings of 7–9 for the same candidate QI for a panel size of 14 in accordance with RAND/UCLA methodology [18]. Experts were asked to provide free-text comments to list additional QIs that should be considered at future expert panel discussions. To standardize their familiarity with evidence-based recommendations, expert panel members were provided with a supplemental literature review report that displayed all candidate QIs, organized by domain, along with their associated guidelines recommendations, strength of recommendation, joints, and comorbidities (Additional File 2).

### Expert panel discussion

Expert panel members convened via the health system’s tele-conferencing platform to discuss survey results, preliminary categories, and the supplemental literature review report. Experts discussed their interpretation of the literature, their insights from clinical practice, and compared the merits of a QI versus other QIs under consideration. After discussing an individual QI during a meeting, all experts in attendance voted to include or exclude that QI. Quality indicators that received ≥ 8 include votes (58% of 14 voting members) were considered valid and feasible and were advanced to priority ranking.

### Priority ranking

Priority was evaluated using an electronic survey to score each valid and feasible QI using a 9-point Likert scale (with 1 indicating lowest priority and 9 indicating highest priority).

### Data management and analysis

Extraction of guideline recommendations from literature search and distribution of electronic surveys for feasibility screening, validity screening, and priority ranking were managed using Research Electronic Data Capture (REDCap) electronic data capture tools [23]. All statistical analyses were performed using STATA (version 16, StataCorp LLC, College Station, Texas, United States).

## Results

### Literature search

**Fig. 1:**
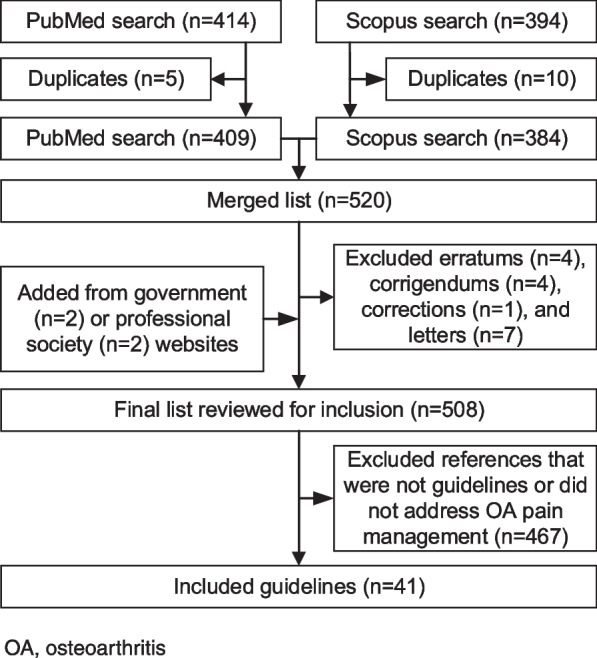
Flow chart of literature search

Of 520 unique references identified from literature search and 4 identified from government/society websites, we included 41 evidence-based guidelines (Fig. 1) [24–64]. We then extracted 741 recommendations. If the guideline presented recommendations in table format, we formulated recommendation sentences using the intervention, strength of recommendation, applicable joints, and applicable comorbidities. Two investigators (Rizk and Swan) collapsed these 741 recommendations into 115 proposed QIs (Supplemental Results 1 section of Additional File 1).

### Feasibility

A subgroup of 5 expert panel members (Fink, Flores, Rizk, Swan, Tajchman) completed the feasibility survey of 115 proposed QIs. The feasibility screen excluded 28 proposed QIs. If the subgroup was uncertain about the feasibility of a proposed QI, the item was retained and advanced to validity screening for further evaluation.

### Face validity

All 14 members of the expert panel completed the face validity survey of 87 candidate QIs that were retained after feasibility screening. Scores from survey responses were used to preliminarily categorize candidate QIs as appropriate (*n* = 22), uncertain (*n* = 40), or inappropriate (*n* = 25).

### Expert panel discussion

**Fig. 2:**
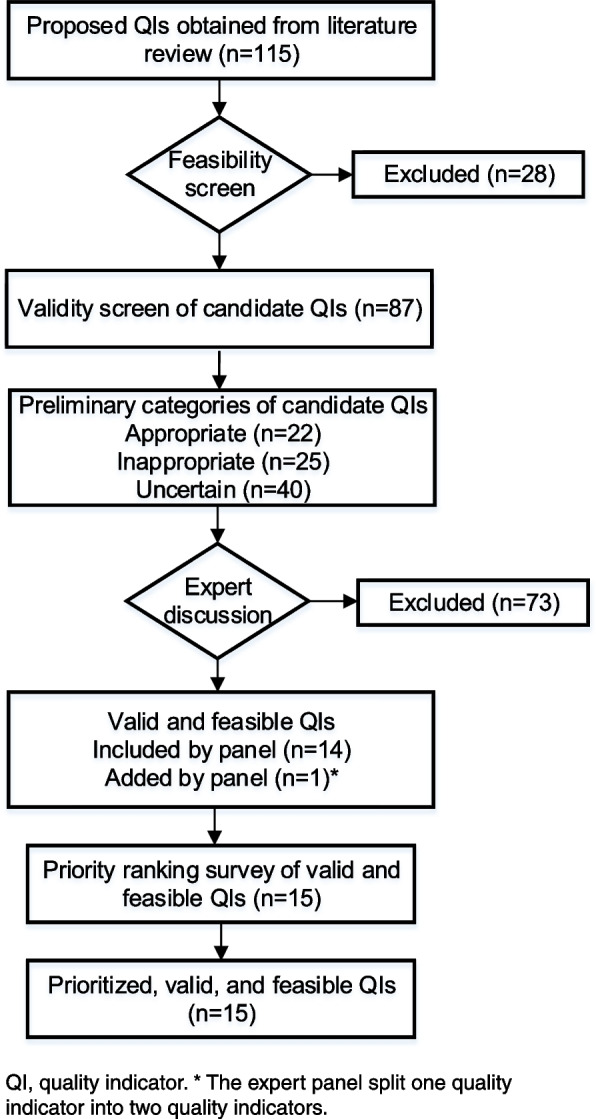
Flow chart of quality indicator inclusion through 5 steps of the expert consensus

The expert panel convened 13 times over a 2-month period (08/2021 to 10/2021). All 14 members participated in expert panel discussions. Of 87 candidate QIs that were discussed, 73 were excluded. The expert panel split one QI into two QIs, both of which were included. This consensus process yielded a total of 15 valid and feasible QIs (Fig. 2). Although candidate QIs were originally worded as non-directional statements (e.g., “proportion of patients on combinations of non-steroidal anti-inflammatory drugs [NSAIDs]”), the panel was instructed to indicate a direction when appropriate (e.g., “avoid combinations of NSAIDs”). The expert panel carefully reviewed clusters of candidate QIs that were similar and looked for opportunities to consolidate key concepts into a unique and non-overlapping set of quality indicators. Additionally, the expert panel reworded QIs to focus on the tasks that are explicitly managed by PCPs (Supplemental Results 2 section of Additional File 1).

### Prioritization

All 14 members of the expert panel completed the prioritization survey of the 15 valid and feasible QIs. Table S-1 (Additional File 1) shows all the ranks and scores used to calculate the final priority ranking. The prioritized list of valid and feasible QIs is shown in Table 1.

**Table 1:** Prioritized list of valid and feasible quality indicators

Rank	Quality Indicator
1	Add a PPI if oral NSAIDs are used in patients with elevated GI risk [24, 25, 27, 30, 31]
2	Avoid oral NSAIDs among patients with CKD [24]
3	Track and minimize opioid use (including tramadol) [24–31, 34, 40, 43, 53, 57]
4	Provide general OA education [24, 25, 27, 28, 30, 31, 38, 41, 46–49, 51, 52, 55, 56]
5	Refer patients for PT/OT [24, 26, 43, 46–48, 57, 58]
6	Use topical NSAIDs for superficial joints (knee, hand, elbow, or foot) [24–32, 40, 51, 52, 55, 57]
7	Use oral NSAIDs [24–31, 48, 51–53, 55, 57, 58]
8	Refer patients with a BMI > 40 kg/m^2^ who have OA in lower extremity joints for weight management (including nutrition management, bariatric surgery, or a metabolic clinic) [24–29, 31, 41, 48, 53, 55, 56]
9	Select naproxen if oral NSAIDs are used in patients with elevated CV risk [27, 30]
10	Avoid combinations of oral NSAIDs [32]
11	Refer patients who fail conservative therapy to a specialist (orthopedic surgery, rheumatology, pain specialist, or physiatry) [24, 27, 28, 30, 31, 36, 50–53, 55]
12	Track and minimize tramadol use [26, 30, 32]
13	Use oral acetaminophen [24–28, 30, 40, 48, 55, 57]
14	Screen for depression and anxiety [29, 56]
15	Avoid unnecessary imaging for OA management [27, 54]

*BMI* Body mass index, *CKD* Chronic kidney disease, *CV* Cardiovascular, *GI* Gastrointestinal, *NSAIDs* Nonsteroidal anti-inflammatory drugs, *OA* Osteoarthritis, *PPI* Proton pump inhibitor, *PT/OT* Physical therapy/occupational therapy

## Discussion

This study established a set of 15 prioritized, valid, and feasible QIs by systematically combining recent scientific evidence from published guidelines with expert opinion from clinical stakeholders.

Over 700 recommendations were extracted from 41 guidelines that were published during the era of acknowledgement of an opioid crisis in the United States. A panel of 14 experts completed surveys and participated in in-depth discussions to evaluate potential merits and limitations of QIs. To ensure buy-in from PCPs, the expert panel considered pragmatic factors of clinic workflow, referral systems, payor models, perceived impact, and patient acceptance/engagement that are relevant for primary care in the United States.

### QIs for safety of OA pain medications

Since the relative effectiveness of medications commonly used to manage OA pain is unclear and may be dose dependent [65], the expert panel prioritized six QIs to optimize safe use of pain medications: add proton pump inhibitor (PPI) to NSAID for patients with gastrointestinal risk (#1), avoid oral NSAIDs in chronic kidney disease (CKD) (#2), minimize opioids (#3), select naproxen if an NSAID is used among patients with cardiovascular risk (#9), avoid combinations of NSAIDs (#10), and minimize tramadol (#12).

Guideline recommendations to reduce the risk of gastrointestinal injury from NSAIDs among at risk patients include use of cyclooxygenase-2 (COX2) selective inhibitors [25, 27], concurrent PPI [24, 25, 27, 30], or use of COX2 selective inhibitors plus concurrent PPI [24, 31]. Although OA guidelines do not explicitly define gastrointestinal risk, the 2009 American College of Gastroenterology guidelines provide a pragmatic definition: age > 65, history of peptic ulcer disease, or concomitant use of aspirin, antiplatelets, anticoagulants, or steroids [66]. The expert panel believed that the addition of the PPI was more important than focusing on selection of an NSAID based on COX1 vs COX2 selectivity. Among patients with elevated risk for cardiovascular side effects from NSAIDs, guidelines suggest that naproxen is allowable, whereas COX2 inhibitors or non-selective NSAIDs may increase risk for cardiovascular adverse events [27, 30]. Although short courses and low doses of NSAIDs may be reasonable for some patients with stage 3 CKD, the expert panel recommended that NSAIDs be avoided for CKD stages 4 and 5 [24]. Because patients with OA may receive NSAID prescriptions from multiple providers (PCPs, rheumatologists, and orthopedic surgeons) and may take over-the-counter NSAIDs, they are at risk for using multiple of NSAIDs simultaneously. Therefore, comprehensive medication reconciliation should be conducted during primary care visits to identify and remove combinations of NSAIDs.

The panel included two QIs focused on opioid use. Several guidelines provide specific recommendations for tramadol [26, 30, 32]. Historically, tramadol was not scheduled by the Drug Enforcement Agency but is currently listed as schedule IV. The expert panel believed that many primary care clinicians may be more comfortable prescribing tramadol compared with other opioids although tramadol 50 mg has a morphine milligram equivalent of 5, which is equivalent to other commonly used opioids (e.g., hydrocodone 5 mg and codeine 30 mg). Therefore, the expert panel believed it was necessary to specifically track and minimize tramadol use. The expert panel chose the phrase “track and minimize” rather than “avoid” for QIs related to opioids as short courses of opioids are appropriate for some patients with advanced OA that is refractory to conventional therapy. The expert panel believed that tracking this QI and reporting this information back to PCPs would facilitate opioid stewardship. Some patients with OA will be taking opioids for other comorbid conditions and matching the opioid prescription to the specific indication through automated alerting or reporting may be challenging. Therefore, it is unreasonable to target 0% for these two QIs.

### QIs for education, weight-management, and psychological wellbeing

Experts included three QIs for providing general OA education (#4), referring patients with BMI > 40 kg/m^2^ and lower extremity OA to weight management (#8), and screening for depression and anxiety (#14). Many guidelines recommend education alone or in combination with other interventions (e.g., exercise or weight management) as a safe and cost-effective intervention. Experts acknowledged that providers may have different styles for delivering education (e.g., verbal counseling, written material, or videos) and documenting in the EHR that this education was provided. Therefore, the overall prioritization of this QI was slightly reduced due to resources that would be needed to standardize workflow among PCPs for documenting that education was provided. Experts envisioned that an educational packet could be loaded into the EHR, printed in the after-visit summary, and posted in the patient-facing medical record portal. Although experts excluded QIs related to exercise, transcutaneous electrical nerve stimulation, and weight management if BMI > 25 kg/m^2^, these could be included in educational material provided to patients.

Weight management is an evidence-based strategy to reduce stress on joints in the lower extremities among obese OA patients. The expert panel believed that BMI thresholds of > 25 kg/m^2^ (overweight) or > 30 kg/m^2^ (obese) from guidelines would include a large volume of OA patients due to high prevalence of obesity in our community [28, 29, 31, 41, 53, 55], and that a higher threshold > 40 kg/m^2^ would be more appropriate to trigger referral. Although some PCPs provide weight management services for patients with a BMI < 40 kg/m^2^ as part of their practice, the expert panel expected PCPs to refer patients with a BMI > 40 kg/m^2^ for weight management interventions.

Primary care physicians are strategically positioned to screen for depression and anxiety. This is feasible at our health system since a quick and simple Patient Health Questionnaire 2 depression screening tool is already available within ambulatory visits in our EHR [67].

### QIs for optimizing first-line medications

The expert panel included QIs that promote use of topical NSAIDs for superficial joints (#6), systemic/oral NSAIDs (#7), and acetaminophen (#13). Given their relatively low cost, ease of access, and acceptable safety profiles, these medications are commonly considered as first-line pharmacological therapies for OA pain management in OA guidelines. Although some OA guidelines [24, 32, 36] and previous lists of QIs [68–70] emphasized the use of these medications as first-line medication therapy, the expert panel believe that it would not always be feasible for quality analysts to establish the sequence of medications previously trialed when calculating these QIs, especially for patients with long histories of OA or multiple prescribing providers. Even though these therapies may not be effective monotherapies for all patients, they can be used in combination across the continuum of OA severity. Therefore, our QIs focus on the proportion of patients (or clinic visits) that are receiving these therapies at a given time (point prevalence). Although the goal is not to achieve 100% due to contraindications and treatment failure, these QIs can be used to track changes in prescribing patterns over time and evaluate the impact of targeted quality initiatives. Another potential use of these QIs is to compare the relative exposure of these therapies against exposure to opioids in a population over time. One limitation identified by the expert panel is that use of over-the-counter formulations of acetaminophen, topical NSAIDs, and oral NSAIDs may be underreported in the EHR since prescriptions are not required.

### QIs for referral

The expert panel believed that most patients with OA can benefit from and should be referred to physical therapy/occupational therapy (#5). The expert panel believed that PCPs should refer patients who fail conservative therapy to a specialist (#11). Conservative therapy could be pharmacological or non-pharmacological and can be provided by a PCP. Candidate QIs regarding choice of intraarticular injection or surgical approach (e.g., arthroscopic procedure) are not made by the PCP at the time of referral and were therefore excluded from the list of final QIs.

### QIs for imaging

The expert panel recommended that PCPs avoid unnecessary imaging for OA management (#15). Specifically, imaging for evaluating the effect of pharmacological or non-pharmacological interventions would be unnecessary. At our health-system, physician orders for imaging are associated with diagnosis codes which allow for feasible evaluation of this QI. Experts believed that streamlined coordination (e.g., consensus order panels) between PCPs and the surgeons could reduce unnecessary imaging. This QI does not include imaging related to the original diagnosis of OA or a substantial change in clinical status.

### Limitations

Although the multi-disciplinary expert panel represents clinicians with a variety of backgrounds who received training at diverse institutions, all experts were recruited from a single health-system. The experts were asked to evaluate feasibility regarding the current status of care at our health-system, and it is possible that some QIs that are not feasible at our health-system may be feasible at other health-systems. As a pragmatic modification of the RAND/UCLA approach, our expert discussions occurred over multiple 60/90-min meetings rather than a focused workshop on 1–2 days. Some experts were not available for some discussions. Relevant clinical guidelines may have been published before or after the time frame of January 2015 to March 2021 that was used for the literature search. Patients with osteoarthritis were not invited to participate in the expert panel for this study.

## Conclusion

A multi-professional expert panel engaged in a consensus strategy that was guided by literature to develop a set of 15 prioritized, valid, and feasible QIs that can be used to track quality initiatives for OA pain management in the primary care setting. Future research is needed to develop operational definitions to measure and track each QI using structured data in the EHR. Additionally, future studies should evaluate associations between these QIs and important health outcomes.

## Supplementary Information

**Additional file 1. **

**Additional file 2.**

## Data Availability

All data generated or analyzed during this study are included in this published article and its supplementary information files.

## Abbreviations

BMI: Body mass index
CKD: Chronic kidney disease
CV: Cardiovascular
EHR: Electronic health record
GI: Gastrointestinal
NSAIDs: Nonsteroidal anti-inflammatory drugs
OA: Osteoarthritis
PPI: Proton pump inhibitor
PT/OT: Physical therapy/occupational therapy
PCPs: Primary care physicians
QI: Quality indicator
RAND/UCLA: Research and Development Corporation/University of California Los Angeles
REDCap: Research Electronic Data Capture
