# Supplementary material for: Quality indicators for osteoarthritis pain management in the primary care setting
Source: BMC Musculoskelet Disord. 2023 Jun 30;24:538. doi: 10.1186/s12891-023-06637-x (PMC10311862; doi:10.1186/s12891-023-06637-x)
Supplement: Supplementary file 1 — Additional file 1. [file 12891_2023_6637_MOESM1_ESM.docx]

**Additional File 1**

**Supplementary Methods 1.** Search terms used to identify publish guidelines for the treatment of osteoarthritis

| **Source** | **Search term** |
| --- | --- |
| PubMed | ("Osteoarthritis"[Title/Abstract] OR "Osteoarthritis"[MeSH Terms]) AND ("guideline"[Publication Type] OR "practice guideline"[Publication Type] OR ("Guidelines as Topic"[MeSH Terms] OR "Practice Guidelines as Topic"[MeSH Terms]) OR ("guideline*"[Title] OR "position statement*"[Title] OR "consensus*"[Title] OR "best practice*"[Title] OR "recommendation*"[Title] OR "guidance"[Title])) AND "english"[Language] AND 2015/01/01:2021/12/31[Date - Publication] |
| Scopus | TITLE-ABS-KEY (osteoarthritis) AND TITLE (guideline OR "position statement*" OR consensus* OR "best practice*" OR recommendation* OR guidance) AND LANGUAGE (english) AND PUBYEAR > 2014 |

**Supplemental Results 1.**

The following example illustrates the process by which investigators collapsed 741 recommendations into 115 proposed QIs. The following three recommendations that were identified for tramadol were all consolidated into one proposed QI: “proportion using tramadol”:

1. “Tramadol is conditionally recommended for patients with knee, hip, and/or OA.”^1^
2. “The use of tramadol in the case of severe pain in its various administration forms is recommended.”^2^
3. “The short-term use of tramadol may be considered for severely symptomatic OA patients and there is good evidence that tramadol works if prescribed properly.”^3^

**References**

1. Kolasinski SL, Neogi T, Hochberg MC, et al. 2019 American College of Rheumatology/Arthritis Foundation Guideline for the Management of Osteoarthritis of the Hand, Hip, and Knee. Arthritis care & research. 2020;72(2):149-162.
2. Rillo O, Riera H, Acosta C, et al. PANLAR Consensus Recommendations for the Management in Osteoarthritis of Hand, Hip, and Knee. J Clin Rheumatol. 2016;22(7):345-354.
3. Bruyere O, Cooper C, Pelletier JP, et al. A consensus statement on the European Society for Clinical and Economic Aspects of Osteoporosis and Osteoarthritis (ESCEO) algorithm for the management of knee osteoarthritis-From evidence-based medicine to the real-life setting. Semin Arthritis Rheum. 2016;45(4 Suppl):S3-11.

**Supplemental Results 2**

The following examples illustrates the process by which the expert panel reworded QIs to focus on the tasks that are explicitly managed by PCPs. Candidate QI of “proportion receiving psychological care” is not entirely under the control of PCPs, since some patients are referred to a specialist for psychological care. Therefore, the expert panel reworded this candidate QI to “screen for anxiety and depression,” which was considered an expectation of PCPs during primary care visits.

**Table S-1.** Scores and ranks for the final list of quality indicators

| **Quality Indicator** | **Mean**  **(Rank)** | **Median (Rank)** | **25^th^ percentile**  **(Rank)** | **75^th^ percentile (Rank)** | **Sum of ranks*** | **Final rank** |
| --- | --- | --- | --- | --- | --- | --- |
| Add a PPI if oral NSAIDs are used in patients with elevated GI risk | 7.71 (1) | 8.00 (2.5) | 7.00 (2.5) | 9.00 (4) | 10.0 | 1 |
| Avoid oral NSAIDs among patients with CKD | 7.64 (2) | 8.00 (2.5) | 7.00 (2.5) | 9.00 (4) | 11.0 | 2 |
| Track and minimize opioid use (including tramadol) | 7.21 (5) | 7.50 (4.5) | 7.00 (2.5) | 9.00 (4) | 16.0 | 3 |
| Provide general OA education | 7.36 (4) | 8.50 (1) | 5.00 (9) | 9.00 (4) | 18.0 | 4 |
| Refer patients for PT/OT | 7.57 (3) | 7.50 (4.5) | 7.00 (2.5) | 8.00 (10.5) | 20.5 | 5 |
| Use topical NSAIDs for superficial joints (knee, hand, elbow, or foot) | 6.79 (6) | 7.00 (8) | 5.00 (9) | 9.00 (4) | 27.0 | 6 |
| Use oral NSAIDs | 6.57 (8.5) | 6.50 (11.5) | 5.00 (9) | 9.00 (4) | 33.0 | 7 |
| Refer patients with a BMI>40 who have OA in lower extremity joints for weight management (including nutrition management, bariatric surgery, or a metabolic clinic) | 6.71 (7) | 7.00 (8) | 5.00 (9) | 8.00 (10.5) | 34.5 | 8 |
| Select naproxen if oral NSAIDs are used in patients with elevated CV risk | 6.36 (10.5) | 6.50 (11.5) | 5.00 (9) | 9.00 (4) | 35.0 | 9 |
| Avoid combinations of oral NSAIDs | 6.57 (8.5) | 7.00 (8) | 5.00 (9) | 8.00 (10.5) | 36.0 | 10 |
| Refer patients who fail conservative therapy to a specialist (orthopedic surgery, rheumatology, pain specialist, or physiatry) | 6.36 (10.5) | 7.00 (8) | 5.00 (9) | 8.00 (10.5) | 38.0 | 11 |
| Track and minimize tramadol use | 6.07 (12) | 7.00 (8) | 5.00 (9) | 7.00 (14) | 43.0 | 12 |
| Use oral acetaminophen | 5.86 (13) | 5.00 (13.5) | 5.00 (9) | 8.00 (10.5) | 46.0 | 13 |
| Screen for depression and anxiety | 5.57 (14) | 5.00 (13.5) | 4.00 (14) | 8.00 (10.5) | 52.0 | 14 |
| Avoid unnecessary imaging for OA management | 4.43 (15) | 4.50 (15) | 2.00 (15) | 6.00 (15) | 60.0 | 15 |

BMI, body mass index; CKD, chronic kidney disease; CV, cardiovascular; GI, gastrointestinal; NSAIDs, nonsteroidal anti-inflammatory drugs; OA, osteoarthritis; PPI, proton pump inhibitor; PT/OT, physical therapy/occupational therapy

* Survey results were analyzed by calculating 4 summary statistics for each QI: mean, median, 25th percentile, and 75th percentile. Each summary statistic was used to create a priority rank (e.g., QIs were sorted by mean and assigned ranks). Because each individual rank method resulted in multiple ties, a final rank score was calculated for each QI as the sum of the 4 individual rank scores. The final priority ranking was based on the final rank score.
