# Supplementary material for: Quality indicators for osteoarthritis pain management in the primary care setting
Source: BMC Musculoskelet Disord. 2023 Jun 30;24:538. doi: 10.1186/s12891-023-06637-x (PMC10311862; doi:10.1186/s12891-023-06637-x)
Supplement: Supplementary file 2 — Additional file 2. [file 12891_2023_6637_MOESM2_ESM.pdf]

Supplemental Report for Validity Screen

Report prepared by Joshua Swan, PharmD, MPH, FCCM, BCPS

Contents

Abbreviations 4
Domain: 01 Topical 5
QI v1_1: proportion using topical NSAIDs 5
QI v1_2: proportion using topical capsaicin 7
Domain: 02 Intra-articular 8
QI v2_1: IA PRP Technique: Proportion on concurrent antiplatelet therapy 8
QI v2_2: IA PRP Technique: Proportion with >3 injections 9
QI v2_3: IA PRP Technique: Proportion with anti-inflamatory medications in days before or after injection 10
QI v2_4: IA PRP Technique: Proportion with bloodcount labs within 3 months prior 11
QI v2_5: IA PRP Technique: Proportion with ultrasound guidance 12
QI v2_6: IA PRP Technique: Proportion with volume that is 4 - 8 mL 13
QI v2_7: IAHA technique: Proportion with ultrasound guidance if iodine allergy 14
QI v2_8: prop of IA injections performed with imaging 15
QI v2_9: proportion using IA Botulinum tox 16
QI v2_10: proportion using IA Dextrose prolothera 17
QI v2_11: proportion using IA PRP 18
QI v2_12: proportion using IA Prolotherapy 19
QI v2_13: proportion using IA stem cells 20
QI v2_14: proportion using IA triamcinolone ER (FX006) 21
QI v2_15: proportion using IACS 22
QI v2_16: proportion using IAHA 24
QI v2_17: proportion using combo of IACS/IAHA 26
QI v2_18: proportion with coadministration of IACS and PRP 27
Domain: 03 Biologics 28
QI v3-1: proportion using Biologics 28
Domain: 04 Systemic 29
QI v4_1: proportion using IM steroids 29
QI v4_2: proportion using NSAIDs 30
QI v4_3: proportion using NSAIDs + PPI 31
QI v4_4: proportion using acetaminophen 32
QI v4_5: proportion using bisphosphonates 33
QI v4_6: proportion using calcitonin 34
QI v4_7: proportion using colchicine 35
QI v4_8: proportion using combinations of NSAIDs 36
QI v4_9: proportion using cox-2 inhibitors 37

QI v4_10: proportion using cox-2 inhibitors + PPI 38
QI v4_11: proportion using doxycycline 39
QI v4_12: proportion using duloxetine 40
QI v4_13: proportion using hydroxychloroquine 41
QI v4_14: proportion using methotrexate 42
QI v4_15: proportion using naproxen 43
QI v4_16: proportion using naproxen + PPI 44
QI v4_17: proportion using non-selective NSAIDs 45
QI v4_18: proportion using non-selective NSAIDs + PPI 46
QI v4_19: proportion using opioids 47
QI v4_20: proportion using oral steroids 49
QI v4_21: proportion using pregabalin and gabapentin 50
QI v4_22: proportion using statins 51
QI v4_23: proportion using sustained-release tramadol 52
QI v4_24: proportion using synthetic DMARDs 53
QI v4_25: proportion using tramadol 54
Domain: 05 Support devices 55
Domain: 06 Supplement 56
QI v6_1: proportion using chondroitin 56
QI v6_2: proportion using chondroitin + glucosamine 57
QI v6_3: proportion using collagen 58
QI v6_4: proportion using combination formulation of chondroitin and glucosamine 59
QI v6_5: proportion using glucosamine 60
QI v6_6: proportion using methylsulfonylmethane 61
QI v6_7: proportion using omega 3/6 fatty acids 62
QI v6_8: proportion using oral hyaluronic acid 63
QI v6_9: proportion using prescription chondroitin sulfate 64
QI v6_10: proportion using vitamin D 65
Domain: 07 Alternative therapy 66
QI v7_1: proportion using TENS 66
QI v7_2: proportion using acupuncture 67
QI v7_3: proportion using electrical stimulation (TENS, inferential, neuromuscular ENS, or pulsed/non-pulsed muscle stimulation) 68
QI v7_4: proportion using electromagnetic therapy 69
QI v7_5: proportion using iontophoresis 70
QI v7_6: proportion using kinesio taping 71
QI v7_7: proportion using laser therapy 72
QI v7_8: proportion using nerve block therapy 73
QI v7_9: proportion using pulsed vibration therapy 74
QI v7_10: proportion using radiofrequency (shockwave or ablation) 75
QI v7_11: proportion using therapeutic ultrasound 76
Domain: 08 Education 77
QI v8_1: proportion receiving education 77
Domain: 09 Behavioral/psychosocial 78
QI v9_1: proportion receiving psychological care 78
QI v9_2: proportion receiving vocational rehabilitation 79
QI v9_3: proportion using Cognitive Behavioral Therapy 80
Domain: 10 Procedures 81
QI v10_1: proportion referred to arthroscopic procedures 81
QI v10_2: proportion referred to surgery 82
Domain: 11 Other 83

QI v11_1: proportion receiving an annual evaluation 83
QI v11_2: proportion receiving routine imaging to monitor OA 84
QI v11_3: proportion using ultrasound to monitor OA 85
Domain: 12 Exercise 86
QI v12_1: proportion receiving exercise + education 86
QI v12_2: proportion receiving mobilization and manipulation 87
QI v12_3: proportion receiving weight management 88
QI v12_4: proportion receiving weight management + exercise 89
QI v12_5: proportion using Cognitive Behavioral Therapy + exercise 90
QI v12_6: proportion using aquatic exercise 91
QI v12_7: proportion using exercise 92
QI v12_8: proportion using land-based exercise 94
QI v12_9: proportion using mind-body exercise (Yoga or Tai Chi) 96
QI v12_10: proportion using minescectomy 97
QI v12_11: proportion using physical therapy 98
References 100

Abbreviations

Abbreviations for joints: poly = polyarticular; shldr = shoulder
Abbreviations for comorbidities: CV = cardiovascular; GI = gastrointestinal; WPD = widespread pain/depression
Level of recommendation (LVL): Str = strong; Con = conditional; NS = not specified

Domain: 01 Topical

QI v1_1: proportion using topical NSAIDs

Comment: Not applicable for hip, poly, or widespread pain

id	verbatim_guideline_rec	LVL	Knee	Hip	Hand	Poly	Shldr	CV	GI	Frail	WPD
1-10	Topical NSAIDs are strongly recommended for patients with knee OA and conditionally recommended for patients with hand OA.	Str	1	.	.	.	.	.	.	.	.
1-37	Topical NSAIDs are strongly recommended for patients with knee OA and conditionally recommended for patients with hand OA.	Cond	.	.	1	.	.	.	.	.	.
18-4	Topical NSAIDs are the first choice of treatment.	NS	.	.	1	.	.	.	.	.	.
2-160	Level 1B/2 conditional recommendation for Topical NSAIDs	Cond	.	.	.	1	.	1	1	1	.
2-161	Level 4A conditional recommendation against Topical NSAIDs	Cond	.	.	.	1	.	.	.	.	1
2-4	Level 1A strong recommendation for topical NSAIDs	Str	1	.	.	.	.	1	1	1	.
2-5	Level 2 conditional recommendation for topical NSAIDs	Cond	1	.	.	.	.	.	.	.	1
2-97	Level 3/4A conditional recommendation against Topical NSAIDs	Cond	.	1	.	.	.	.	.	.	.
22-4	We recommend offering topical non-steroidal anti-inflammatory drugs for patients with pain associated with osteoarthritis of the knee.	Str	1	.	.	.	.	.	.	.	.
24-2	Topical treatments are preferred over systemic treatments because of safety reasons. Topical NSAIDs are the first pharmacological topical treatment of choice	NS	.	.	1	.	.	.	.	.	.
28-24	Topical NSAIDs may be indicated in patients with gastrointestinal risk, even though the analgesic response decreases after 1 year of use	Str	1	.	.	.	.	.	1	.	.
28-35	Topical NSAIDs are indicated as being effective and safe for mild to moderate pain in patients with few affected joints and in elderly patients with mild to moderate persistent pain.	Str	.	.	1	.	.	.	.	.	.
3-14	Patients with age >75 years should use topical rather than oral NSAIDs, even though the analgesic response decreases after 1 year of use.	NS	1	1	1	.	.	.	.	.	.
30-3	Topical NSAIDs can be proposed (Level 1A, Strength B)	Cond	1	.	.	.	.	.	.	.	.
31-12	Topical NSAIDs can be used in patients who have mild disease with intermittent pain, short-term stiffness after inactivation and radiological knee OA of grade 0-1.	NS	1	.	.	.	.	1	1	.	.
35-9	For patients with mild pain, topical application of non-steroidal anti-inflammatory drugs (NSAIDs) is recommended to reduce local pain (1B)	Str	1	1	1	.	.	.	.	.	.
4-10	A strong recommendation to the use of topical NSAIDs as cyclic add-on analgesia in Step 1, for patients who are still symptomatic after the use of Step 1 background therapy, and prior to use of oral NSAIDs.	Str	1	.	.	.	.	.	.	.	.
40-19	Consider topical NSAIDs for pain relief in addition to core treatments for people with knee or hand osteoarthritis. Consider topical NSAIDs and/or paracetamol ahead of oral NSAIDs, COX-2 inhibitors or opioids	NS	1	.	1	.	.	.	.	.	.
41-2	Topical or oral non-steroid anti-inflammatory drugs (NSAIDS) are recommended for the treatment of pain in patients waiting for surgery (LE 1a; LR A; LA 82%)	Str	1	1	.	.	.	.	.	.	.
8-13	Topical NSAIDs may be considered as the preferred treatment option, particularly in OA patients aged 75 years or	NS	1	.	.	.	.	1	1	.	.

	older, and those with co-morbidities, or those at an increased risk of GI, ardiovascular (CV), or renal side effects.										
8-14	Topical non-steroidal anti-inflammatory drugs (NSAIDs) may provide additional symptomatic treatment with the same degree of efficacy as oral NSAIDs without the systemic safety concerns.	NS	1	.	.	.	.	.	.	.	.

QI v1_2: proportion using topical capsaicin

Comment: 6 for & 6 against; usually recommended for knee

id	verbatim_guideline_rec	LVL	Knee	Hip	Hand	Poly	Shldr	CV	GI	Frail	WPD
1-32	Topical capsaicin is conditionally recommended for patients with knee OA and conditionally recommended against in patients with hand OA.	Cond	1	.	.	.	.	.	.	.	.
1-41	Topical capsaicin is conditionally recommended for patients with knee OA and conditionally recommended against in patients with hand OA.	Cond	.	.	1	.	.	.	.	.	.
2-148	Level 5 strong recommendation against Topical Capsaicin	Str	.	1	.	.	.	.	.	.	.
2-221	Level 5 strong recommendation against Topical Capsaicin	Str	.	.	.	1	.	.	.	.	.
2-28	Level 4B/4A conditional recommendation against Topical Capsaicin	Cond	1	.	.	.	.	.	.	.	.
22-6	We suggest offering topical capsaicin for patients with pain associated with osteoarthritis of the knee.	Cond	1	.	.	.	.	.	.	.	.
28-59	Capsaicin gel was shown to be an effective treatment for knee OA accompanied by mild to moderate pain	Cond	1	.	.	.	.	.	.	.	.
3-20	Topical pharmacological treatments are preferred over systemic treatments, especially for mild to moderate pain and when only a few joints are affected. Topical NSAIDs and capsaicin gel are effective and safe treatments.	NS	1	1	1	.	.	.	.	.	.
30-10	Low-dose topical capsaicin (< 1%) can be considered (Level 1A, Strength C)	NS	1	.	.	.	.	.	.	.	.
36-54	We suggest not offering topical capsaicin for people with knee OA	Cond	1	.	.	.	.	.	.	.	.
40-20	Topical capsaicin should be considered as an adjunct to core treatments for knee or hand osteoarthritis	NS	1	.	1	.	.	.	.	.	.
40-21	Do not offer rubefacients for treating osteoarthritis	NS	1	.	1	.	.	.	.	.	.

Domain: 02 Intra-articular

QI v2_1: IA PRP Technique: Proportion on concurrent antiplatelet therapy

Comment:

id	verbatim_guideline_rec	LVL	Knee	Hip	Hand	Poly	Shldr	CV	GI	Frail	WPD
19-17	Antiplatelet aggregation therapy is not a contraindication to PRP injections but may alter its efficacy by preventing platelet activation	NS	1	.	.	.	.	.	.	.	.

QI v2_2: IA PRP Technique: Proportion with >3 injections

Comment:

id	verbatim_guideline_rec	LVL	Knee	Hip	Hand	Poly	Shldr	CV	GI	Frail	WPD
19-20	A PRP treatment sequence in knee osteoarthritis may include 1- 3 injections.	NS	1	.	.	.	.	.	.	.	.

QI v2_3: IA PRP Technique: Proportion with anti-inflamatory medications in days before or after injection

Comment:

id	verbatim_guideline_rec	LVL	Knee	Hip	Hand	Poly	Shldr	CV	GI	Frail	WPD
19-2	Anti-inflammatory treatment should be avoided in the days before and after platelet-rich plasma (PRP) treatment	NS	1	.	.	.	.	.	.	.	.

QI v2_4: IA PRP Technique: Proportion with bloodcount labs within 3 months prior

Comment:

id	verbatim_guideline_rec	LVL	Knee	Hip	Hand	Poly	Shldr	CV	GI	Frail	WPD
19-19	A blood count should be obtained less than 3 months before PRP treatment	NS	1	.	.	.	.	.	.	.	.

QI v2_5: IA PRP Technique: Proportion with ultrasound guidance

Comment:

id	verbatim_guideline_rec	LVL	Knee	Hip	Hand	Poly	Shldr	CV	GI	Frail	WPD
19-5	Intra-articular platelet-rich plasma (PRP) knee injections should be performed under ultrasound or fluoroscopic guidance	NS	1	.	.	.	.	.	.	.	.

QI v2_6: IA PRP Technique: Proportion with volume that is 4 - 8 mL

Comment:

id	verbatim_guideline_rec	LVL	Knee	Hip	Hand	Poly	Shldr	CV	GI	Frail	WPD
19-1	The volume of a platelet-rich plasma (PRP) injection in knee osteoarthritis should be 4-8 mL	NS	1	.	.	.	.	.	.	.	.

QI v2_7: IAHA technique: Proportion with ultrasound guidance if iodine allergy

Comment:

id	verbatim_guideline_rec	LVL	Knee	Hip	Hand	Poly	Shldr	CV	GI	Frail	WPD
15-15	When imaging guidance is needed we recommend use ultrasound guidance rather than fluoroscopy in patients with iodine allergy.	Str	1	1	.	.	1	.	.	.	.

QI v2_8: prop of IA injections performed with imaging

Comment: 3 for; 3 hand; 2 hip; 3 knee; 1 shoulder; 1 foot; 1 elbow

id	verbatim_guideline_rec	LVL	Knee	Hip	Hand	Poly	Shldr	CV	GI	Frail	WPD
26-2	Ultrasound guidance may improve accuracy of articular and periarticular injections or aspirations, and it is particularly recommended in structures difficult to access	NS	1	1	1	.	1	.	.	.	.
29-4	The accuracy of intra-articular injection depends on the joint and on the skills of the practitioner and imaging may improve accuracy. Imaging is particularly recommended for joints that are difficult to access due to factors including site (eg, hip), degree of deformity and obesity.	NS	1	.	1	.	.	.	.	.	.
3-2	The accuracy of intra-articular injection depends on the joint and on the skills of the practitioner. Ultrasound-guidance may improve accuracy and it is particularly recommended for joints that are difficult to access due to the site itself, degree of deformity or obesity	NS	1	1	1	.	.	.	.	.	.

QI v2_9: proportion using IA Botulinum tox

Comment: 1 against; joints 1 knee, 1 hip

id	verbatim_guideline_rec	LVL	Knee	Hip	Hand	Poly	Shldr	CV	GI	Frail	WPD
1-23	Intraarticular botulinum toxin injections are conditionally recommended against in patients with knee and/or hip OA.	Cond	1	1	.	.	.	.	.	.	.

QI v2_10: proportion using IA Dextrose prolothera

Comment: 4 against; joints 2 knee, 2 hip, 1 poly

id	verbatim_guideline_rec	LVL	Knee	Hip	Hand	Poly	Shldr	CV	GI	Frail	WPD
2-129	Level 5 strong recommendation against Dextrose Prolotherapy	Str	.	1	.	.	.	.	.	.	.
2-204	Level 5 strong recommendation against Dextrose Prolotherapy	Str	.	.	.	1	.	.	.	.	.
2-62	Level 5 strong recommendation against Dextrose Prolotherapy	Str	1	.	.	.	.	.	.	.	.
36-62	We suggest not offering dextrose prolotherapy for people with knee and/or hip OA	Cond	1	1	.	.	.	.	.	.	.

QI v2_11: proportion using IA PRP

Comment: 8 for & 9 against; joints 14 knee, 3 hip, 1 poly, 1 shoulder

id	verbatim_guideline_rec	LVL	Knee	Hip	Hand	Poly	Shldr	CV	GI	Frail	WPD
1-29	Platelet-rich plasma treatment is strongly recommended against in patients with knee and/or hip OA.	Str	1	1	.	.	.	.	.	.	.
19-10	Intra-articular PRP injections in knee osteoarthritis are a locally well-tolerated treatment	NS	1	.	.	.	.	.	.	.	.
19-13	PRP treatment should be offered as a second line of treatment after failure of oral or non-pharmacological treatment for knee osteoarthritis.	NS	1	.	.	.	.	.	.	.	.
19-14	Intra-articular PRP injections in knee osteoarthritis are a systemically well-tolerated treatment	NS	1	.	.	.	.	.	.	.	.
19-15	Recent neoplasia (malignant tumors, hematological diseases) may be a contraindication to intra-articular PRP injections	NS	1	.	.	.	.	.	.	.	.
19-18	Intra-articular injections of PRP may be useful in severe knee osteoarthritis	NS	1	.	.	.	.	.	.	.	.
19-3	platelet-rich plasma (PRP) treatment should not be used during a flare-up of knee osteoarthritis	NS	1	.	.	.	.	.	.	.	.
19-6	Leucocyte-poor platelet-rich plasma (PRP) should be preferred in knee osteoarthritis	NS	1	.	.	.	.	.	.	.	.
19-7	Intra-articular injections of PRP are an effective symptomatic treatment for early to moderate knee osteoarthritis.	NS	1	.	.	.	.	.	.	.	.
19-8	Symptomatic bilateral knee osteoarthritis can be treated at the same time	NS	1	.	.	.	.	.	.	.	.
2-142	Level 5 strong recommendation against PRP	Str	.	1	.	.	.	.	.	.	.
2-216	Level 5 strong recommendation against PRP	Str	.	.	.	1	.	.	.	.	.
2-73	Level 5 strong recommendation against PRP	Str	1	.	.	.	.	.	.	.	.
28-40	Intra-articular injection of platelet-rich plasma may help to relieve pain associated with knee OA; however, our recommendation is to conduct better quality studies	Cond	1	.	.	.	.	.	.	.	.
3-5	it is unclear if intra-articular injection of mesenchymal stem cells or platelet-rich plasma can help to relieve pain associated with knee OA	NS	1	.	.	.	.	.	.	.	.
34-3	In the absence of reliable evidence, it is the opinion of the work group that injectable biologics, such as stem cells or platelet-rich plasma, cannot be recommended in the treatment of glenohumeral osteoarthritis.	Cond	.	.	.	.	1	.	.	.	.
7-1	Biologic therapies, including stem cell and platelet-rich plasma (PRP) injections, cannot be currently recommended for the treatment of advanced hip or knee arthritis.	NS	1	1	.	.	.	.	.	.	.

QI v2_12: proportion using IA Prolotherapy

Comment: 1 against; joints 1 knee, 1 hip

id	verbatim_guideline_rec	LVL	Knee	Hip	Hand	Poly	Shldr	CV	GI	Frail	WPD
1-34	Prolotherapy is conditionally recommended against in patients with knee and/or hip OA.	Cond	1	1	.	.	.	.	.	.	.

QI v2_13: proportion using IA stem cells

Comment: 4 for & 10 against; joints 11 knee, 4 hip, 1 poly, 1 shoulder

id	verbatim_guideline_rec	LVL	Knee	Hip	Hand	Poly	Shldr	CV	GI	Frail	WPD
1-39	Stem cell injections are strongly recommended against in patients with knee and/or hip OA.	Str	1	1	.	.	.	.	.	.	.
17-8	There is insufficient evidence to support the use of MSCs or BMAC in the treatment of knee OA. [mesenchymal stem cells (MSCs), bone marrow aspirate concentrate (BMAC)]	NS	1	.	.	.	.	.	.	.	.
2-136	Level 5 strong recommendation against IA Stem Cells	Str	.	1	.	.	.	.	.	.	.
2-209	Level 5 strong recommendation against IA Stem Cells	Str	.	.	.	1	.	.	.	.	.
2-66	Level 5 strong recommendation against IA Stem Cells	Str	1	.	.	.	.	.	.	.	.
22-16	We suggest against stem cell injections (e.g., mesenchymal, adipose-derived, and bone marrow-derived) for the treatment of osteoarthritis of the knee.	Cond	1	.	.	.	.	.	.	.	.
25-1	Based on an extensive review of the literature, there is strong evidence for the safety of bone marrow concentrate (BMC) when performed by trained physicians with the appropriate precautions under image guidance utilizing a sterile technique.	Str	1	.	.	.	.	.	.	.	.
25-2	Statement 2: Assessment of clinical effectiveness based on extensive literature shows emerging evidence for multiple musculoskeletal and spinal conditions. â¢ The evidence is highest for knee osteoarthritis with level II evidence based on relevant systematic reviews, randomized controlled trials and nonrandomized studies. There is level III evidence for knee cartilage conditions.	NS	1	.	.	.	.	.	.	.	.
28-41	Intra-articular injection of mesenchymal stem cells derived from the infrapatellar fat pad may be effective at reducing pain and improving knee function. Level of recommendation: IIIC	Cond	1	.	.	.	.	.	.	.	.
3-5	it is unclear if intra-articular injection of mesenchymal stem cells or platelet-rich plasma can help to relieve pain associated with knee OA	NS	1	.	.	.	.	.	.	.	.
34-3	In the absence of reliable evidence, it is the opinion of the work group that injectable biologics, such as stem cells or platelet-rich plasma, cannot be recommended in the treatment of glenohumeral osteoarthritis.	Cond	.	.	.	.	1	.	.	.	.
35-2	For patients with knee OA who have poor responses with intra-articular injection of HA, stem cell injection may be considered (2D)	NS	1	.	.	.	.	.	.	.	.
36-14	We do not recommend offering stem cell therapy for people with knee and/or hip OA	Str	1	1	.	.	.	.	.	.	.
7-1	Biologic therapies, including stem cell and platelet-rich plasma (PRP) injections, cannot be currently recommended for the treatment of advanced hip or knee arthritis.	NS	1	1	.	.	.	.	.	.	.

QI v2_14: proportion using IA triamcinolone ER (FX006)

Comment: 2 against, 1 hip 1 polyarticular

id	verbatim_guideline_rec	LVL	Knee	Hip	Hand	Poly	Shldr	CV	GI	Frail	WPD
2-150	Level 5 strong recommendation against FX006 over IACS	Str	.	1	.	.	.	.	.	.	.
2-222	Level 5 strong recommendation against FX006 over IACS	Str	.	.	.	1	.	.	.	.	.

QI v2_15: proportion using IACS

Comment: 23 for & 3 against; joints 18 knee, 7 hip, 4 hand, 1 poly

id	verbatim_guideline_rec	LVL	Knee	Hip	Hand	Poly	Shldr	CV	GI	Frail	WPD
1-11	Intraarticular glucocorticoid injections versus other injections are conditionally recommended for patients with knee, hip, and/or hand OA.	Cond	1	1	1	.	.	.	.	.	.
1-20	Intraarticular glucocorticoid injections are strongly recommended for patients with knee and/or hip OA and conditionally recommended for patients with hand OA.	Str	1	1	.	.	.	.	.	.	.
1-3	Ultrasound guidance for intraarticular glucocorticoid injection is strongly recommended for injection into hip joints.	Str	.	1	.	.	.	.	.	.	.
1-30	Intraarticular glucocorticoid injections are strongly recommended for patients with knee and/or hip OA and conditionally recommended for patients with hand OA.	Cond	.	.	1	.	.	.	.	.	.
17-2	Intra-articular corticosteroid injections provide short-term, moderate pain relief and the restoration of function and offer a cost effective treatment option in patients with early knee OA.	NS	1	.	.	.	.	.	.	.	.
17-9	Intra-articular corticosteroid injections provide short-term, moderate pain relief and the restoration of function.	NS	1	.	.	.	.	.	.	.	.
2-13	Level 1B/2 conditional recommendation for IACS	Cond	1	.	.	.	.	.	.	.	.
2-173	Level 4B conditional recommendation against IACS	Cond	.	.	.	1	.	.	.	.	.
2-95	Level 3/4B conditional recommendation against IACS	Cond	.	1	.	.	.	.	.	.	.
22-11	We suggest offering an intra-articular corticosteroid injection for patients with persistent pain due to osteoarthritis of the knee inadequately relieved by other interventions.	Cond	1	.	.	.	.	.	.	.	.
22-12	We suggest offering an intra-articular, image-guided corticosteroid injection for patients with persistent pain due to osteoarthritis of the hip inadequately relieved by other interventions.	Cond	.	1	.	.	.	.	.	.	.
24-4	Intra-articular injections of glucocorticoids should not generally be used in patients with hand OA, but may be considered in patients with painful interphalangeal joints	NS	.	.	1	.	.	.	.	.	.
28-1	The administration of intra-articular steroids may be reasonable for knee OA accompanied by inflammation	Cond	1	.	.	.	.	.	.	.	.
28-12	Steroids or intra-articular hyaluronic acid may be considered for use in the treatment of OA of the symptomatic TMC joint	Cond	.	.	1	.	.	.	.	.	.
28-52	Intra-articular corticosteroid injection (ultrasound-guided) may be beneficial to provide fast pain relief	Cond	1	.	.	.	.	.	.	.	.
3-3	intra-articular corticosteroid injection may be beneficial, providing fast pain relief in patients who suffer painful relapses and who do not respond or have a contraindication to analgesics and NSAIDs	NS	1	1	1	.	.	.	.	.	.
30-2	Intra-articular corticosteroid injections can be proposed (Level 1A, Strength A), especially for inflammatory flare-ups with joint effusion (Level 4, Strength D)	Str	1	.	.	.	.	.	.	.	.
31-15	If there are inflammatory findings and the other treatment options have failed, intraarticular glucocorticoids can be applied, not more than 3 times-a-year, for patients with moderate-severe symptoms, functional capacity of either normal or minimally limited and/or radiologic grade of 2-3	NS	1	.	.	.	.	.	.	.	.
32-1	Infiltration with methylprednisolone acetate (MA) is more effective in relieving pain than triamcinolone acetonide (TA) in patients with knee OA in a maximum of six weeks, and equally effective in the improvement of function in up to 12 weeks	Str	1	.	.	.	.	.	.	.	.
32-3	The use of triamcinolone hexacetonide (TH) may be favored over methylprednisolone acetate (MA) for knee arthritis (RA or OA; 1 and 3 week analysis, respectively) based on the fastest onset of action for pain relief	Str	1	.	.	.	.	.	.	.	.
35-12	For patients of knee OA with persistent or moderate to severe pain, intra-articular injection of glucocorticoids is	Str	1	.	.	.	.	.	.	.	.

	recommended for rapid relief of pain in patients with OA, the injection interval should not be shorter than 4 to 6 months (1B)										
36-27	It may be appropriate to offer an intra-articular corticosteroid injection for some people with knee and/or hip OA for short-term pain relief	Cond	1	1	.	.	.	.	.	.	.
4-27	A weak recommendation to the use of IA corticosteroids, which are more effective than IAHA in the first few weeks of treatment in the same patient population	Cond	1	.	.	.	.	.	.	.	.
40-26	Intra-articular corticosteroid injections should be considered as an adjunct to core treatments for the relief of moderate to severe pain in people with osteoarthritis.	NS	1	.	.	.	.	.	.	.	.
41-5	Intra-articular corticoid injections for patients with arthrosis of the knee are recommended (LE 1a; LR A; LA 70%)	Str	1	.	.	.	.	.	.	.	.
5-3	Strong evidence supports the use of intraarticular corticosteroids to improve function and reduce pain in the short-term for patients with symptomatic osteoarthritis of the hip.	Str	.	1	.	.	.	.	.	.	.

QI v2_16: proportion using IAHA

Comment: 19 for & 10 against; joints 18 knee, 8 hip, 3 hand, 1 poly, 2 shoulder, 1 ankle, 1 temporomadibul, 1 trapeziometacar

id	verbatim_guideline_rec	LVL	Knee	Hip	Hand	Poly	Shldr	CV	GI	Frail	WPD
1-33	Intraarticular hyaluronic acid injections are conditionally recommended against in patients with knee and/or first carpometacarpal (CMC) joint OA and strongly recommended against in patients with hip OA.	Str	.	1	.	.	.	.	.	.	.
1-6	Intraarticular hyaluronic acid injections are conditionally recommended against in patients with knee and/or first carpometacarpal (CMC) joint OA and strongly recommended against in patients with hip OA.	Cond	1	.	1	.	.	.	.	.	.
15-1	We recommend performing viscosupplementation under fluoroscopy or ultrasound guidance	Str	.	1	.	.	1	.	.	.	.
15-7	We recommend administering viscosupplementation (VS) in the knee through a lateral patellofemoral route.	Str	1	.	.	.	.	.	.	.	.
17-10	Intra-articular HA provides improvement in pain, function, and stiffness for up to 26 weeks after an injection in patients with mild to moderate knee OA. It is safe with a low risk of adverse events	NS	1	.	.	.	.	.	.	.	.
17-5	Intra-articular injections of High-molecular weight (HMW) HA provide improved pain relief and the restoration of function compared with placebo and can be considered in patients with mild to moderate knee OA.	NS	1	.	.	.	.	.	.	.	.
17-6	HMW HA is superior to High-molecular weight (HMW) HA, with a treatment effect surpassing the minimal clinically important difference (MCID)	NS	1	.	.	.	.	.	.	.	.
17-7	Highly crosslinked HA is more effective than non-cross linked HA	NS	1	.	.	.	.	.	.	.	.
2-14	Level 1B/2 conditional recommendation for IAHA	Cond	1	.	.	.	.	.	.	.	.
2-174	Level 4B/4A conditional recommendation against IAHA	Cond	.	.	.	1	.	.	.	.	.
2-96	Level 4B/4A conditional recommendation against IAHA	Cond	.	1	.	.	.	.	.	.	.
22-13	We suggest offering intra-articular viscosupplementation injection(s) for patients with persistent pain due to osteoarthritis of the knee inadequately relieved by other interventions.	Cond	1	.	.	.	.	.	.	.	.
22-14	We suggest against the use of intra-articular viscosupplementation injection(s) of the hip.	Cond	.	1	.	.	.	.	.	.	.
28-12	Steroids or intra-articular hyaluronic acid may be considered for use in the treatment of OA of the symptomatic TMC joint	Cond	.	.	1	.	.	.	.	.	.
28-36	Intra-articular injection of hyaluronic acid of different molecular weights has proven to be beneficial in the treatment of knee OA	Cond	1	.	.	.	.	.	.	.	.
28-53	The use of hyaluronic acid may be beneficial and, thus, could help to reduce the NSAID use	Cond	.	1	.	.	.	.	.	.	.
3-24	intra-articular injection of hyaluronic acid of different molecular weights may give symptomatic benefit with low toxicity and could help to reduce NSAID use	NS	1	1	1	.	.	.	.	.	.
30-9	Intra-articular hyaluronic acid injections can be proposed (Level 1A, Strength A) without expecting a chondroprotective effect (Level 1B, Strength B)	Str	1	.	.	.	.	.	.	.	.
31-9	Intraarticular hyaluronic acid may be used even though the evidence about its efficiency is uncertain for patients with moderate-severe symptoms, functional capacity of either normal or minimally limited and/or radiologic grade of 2-3	NS	1	.	.	.	.	.	.	.	.
34-1	Strong evidence supports that there is no benefit to the use of hyaluronic acid in the treatment of glenohumeral joint osteoarthritis.	Str	.	.	.	.	1	.	.	.	.
35-14	For patients of knee OA with persistent or moderate to severe pain, intra-articular injection of HA can be considered to improve the patient's symptoms in the long term and delay the time required for joint replacement (2C)	NS	1	.	.	.	.	.	.	.	.
36-60	We suggest not offering viscosupplementation injection for people with knee OA	Cond	1	.	.	.	.	.	.	.	.

36-61	We do not recommend offering viscosupplementation injection for people with hip OA.	Str	.	1	.	.	.	.	.	.	.
4-29	A weak recommendation to the use of IAHA in patients who have contraindications to NSAIDs, or if the patient is still symptomatic despite the use of NSAIDs.	Cond	1	.	.	.	.	.	.	.	.
40-27	Do not offer intra-articular hyaluronan injections for the management of osteoarthritis	NS	1	.	.	.	.	.	.	.	.
41-4	The use of hyaluronic acid is advised against for these patients (LE 1b; LR A; LA 82%)	Str	1	1	.	.	.	.	.	.	.
5-11	Strong evidence does not support the use of intraarticular hyaluronic acid because it does not perform better than placebo for function, stiffness, and pain in patients with symptomatic osteoarthritis of the hip.	Str	.	1	.	.	.	.	.	.	.
8-10	The ESCEO task force recommends the use of Intra-articular (IA) hyaluronic acid (HA) in knee OA patients with mild-moderate disease, and for more severe patients who are either contraindicated to TKR surgery or wishing to delay the surgical procedure	NS	1	.	.	.	.	.	.	.	.
8-9	Intra-articular (IA) hyaluronic acid (HA) should only be administered in knee OA once the acute inflammatory flare has settled	NS	1	.	.	.	.	.	.	.	.

QI v2_17: proportion using combo of IACS/IAHA

Comment: 1 for; knee only

id	verbatim_guideline_rec	LVL	Knee	Hip	Hand	Poly	Shldr	CV	GI	Frail	WPD
17-4	Intra-articular injections of combined HA and corticosteroids in the setting of knee OA can provide significant improvement in pain outcomes and may provide a more rapid onset and longer duration of action than either therapy alone.	NS	1	.	.	.	.	.	.	.	.

QI v2_18: proportion with coadministration of IACS and PRP

Comment: 2 against both knee

id	verbatim_guideline_rec	LVL	Knee	Hip	Hand	Poly	Shldr	CV	GI	Frail	WPD
19-4	PRP should not be mixed with an anesthetic or intraarticular corticosteroid	NS	1	.	.	.	.	.	.	.	.
19-9	Treatment of knee osteoarthritis with PRP should be done away from an intra-articular injection of a corticosteroid	NS	1	.	.	.	.	.	.	.	.

Domain: 03 Biologics

QI v3-1: proportion using Biologics

Comment: All 13 are against. 6 for knee, 6 hip, 4 hand, 2 poly

id	verbatim_guideline_rec	LVL	Knee	Hip	Hand	Poly	Shldr	CV	GI	Frail	WPD
1-13	Tumor necrosis factor inhibitors and interleukin-1 receptor antagonists are strongly recommended against in patients with knee, hip, and/or hand OA.	Str	1	1	1	.	.	.	.	.	.
18-8	Conventional synthetic or biologic DMARDs should not be used in patients with hand OA.	NS	.	.	1	.	.	.	.	.	.
2-137	Level 5 strong recommendation against IL-1 Receptor Antagonists	Str	.	1	.	.	.	.	.	.	.
2-147	Level 5 strong recommendation against TNF-Î± Inhibitors	Str	.	1	.	.	.	.	.	.	.
2-210	Level 5 strong recommendation against IL-1 Receptor Antagonists	Str	.	.	.	1	.	.	.	.	.
2-220	Level 5 strong recommendation against TNF-Î± Inhibitors	Str	.	.	.	1	.	.	.	.	.
2-67	Level 5 strong recommendation against IL-1 Receptor Antagonists	Str	1	.	.	.	.	.	.	.	.
2-78	Level 5 strong recommendation against TNF-Î± Inhibitors	Str	1	.	.	.	.	.	.	.	.
24-5	Patients with hand OA should not be treated with conventional or biological disease-modifying antirheumatic drugs	NS	.	.	1	.	.	.	.	.	.
28-15	Adalimumab or infliximab are not recommended in patients with secondary or erosive hand	Str	.	.	1	.	.	.	.	.	.
36-10	We do not recommend offering IL-1 inhibitors for people with knee and/or hip OA	Str	1	1	.	.	.	.	.	.	.
36-11	We do not recommend offering FGF (Fibroblast growth factor) for people with knee and/or hip OA	Str	1	1	.	.	.	.	.	.	.
36-57	We suggest not offering NGF for people with knee and/or hip OA	Cond	1	1	.	.	.	.	.	.	.

Domain: 04 Systemic

QI v4_1: proportion using IM steroids

Comment: 1 against; joints 1 hand

id	verbatim_guideline_rec	LVL	Knee	Hip	Hand	Poly	Shldr	CV	GI	Frail	WPD
28-34	Intramuscular steroid is not recommended for patients with symptomatic hand OA	Str	.	.	1	.	.	.	.	.	.

QI v4_2: proportion using NSAIDs

Comment: 14 for & 3 against (kidney/CV risk); joints 13 knee, 9 hip, 8 hand; 3 for patients who failed APAP/topical NSAIDs

id	verbatim_guideline_rec	LVL	Knee	Hip	Hand	Poly	Shldr	CV	GI	Frail	WPD
1-15	Oral NSAIDs are strongly recommended for patients with knee, hip, and/or hand OA.	Str	1	1	1	.	.	.	.	.	.
12-24	A eight-week progressive quadriceps strengthening exercise program with Nonsteroidal anti-inflammatory drugs (NSAIDs) (quadriceps exercises while sitting on a chair or in a supine position using ankle weights) for the management of knee osteoarthritis for pain relief (Visual Analogue Scale (VAS)) at eight weeks end of treatment is suggested.	NS	1	.	.	.	.	.	.	.	.
18-9	Oral NSAIDs should be used for a short term for alleviation of symptoms.	NS	.	.	1	.	.	.	.	.	.
22-8	We suggest offering acetaminophen and/or oral non-steroidal anti-inflammatory drugs for pain associated with osteoarthritis of the hip and knee.	Cond	1	1	.	.	.	.	.	.	.
24-6	Oral analgesics, particularly NSAIDs, should be considered for a limited duration for relief of symptoms	NS	.	.	1	.	.	.	.	.	.
28-8	Oral NSAIDs are recommended at the lowest effective dose and for the shortest time possible if patients present inadequate response to acetaminophen/paracetamol. The high risk associated with gastrointestinal and cardiovascular events should be considered.	Str	.	.	1	.	.	.	.	.	.
3-29	In nephropathic patients the use of NSAID and COX-2 inhibitors should be avoided.	NS	1	1	1	.	.	.	.	.	.
3-7	Oral NSAIDs are recommended at the lowest effective dose and for the shortest duration in patients who respond inadequately to paracetamol. NSAIDs (such as ibuprofen, diclofenac and naproxen) and selective COX-2 inhibitors (including celecoxib and etoricoxib) are indicated in moderate pain. Higher doses of oral NSAIDs may be indicated in more severe pain.	NS	1	1	1	.	.	.	.	.	.
31-8	NSAIDs may be used in case of response to acetaminophen is absent or insufficient for patients with moderate-severe symptoms, functional capacity of either normal or minimally limited and/or radiologic grade of 2-3	NS	1	.	.	.	.	.	.	.	.
35-13	For OA patients with persistent pain or moderate or severe pain, it is recommended to choose oral NSAIDs after risk assessment, and use the lowest effective dose for a short period (1-3 months) alone (1B)	Str	1	1	1	.	.	.	.	.	.
36-24	It may be appropriate to offer oral NSAIDs for some people with knee and/or hip OA	Cond	1	1	.	.	.	.	.	.	.
4-17	As advanced pharmacological management in the persistent symptomatic patient (STEP 2) who did not respond to Step 1 background treatment, use intermittent or longer cycles of oral NSAIDs. If increased CV risk - limit the use of any NSAIDs for a maximum of 7 days for non-selective NSAIDs or celecoxib for a maximum of 30 days	NS	1	.	.	.	.	1	.	.	.
4-23	A strong recommendation to the use of oral NSAIDs (selective or non-selective) as Step 2 therapy, if used only intermittently or for longer cycles	Str	1	.	.	.	.	.	.	.	.
4-26	As advanced pharmacological management in the persistent symptomatic patient (STEP 2) who did not respond to Step 1 background treatment, use intermittent or longer cycles of oral NSAIDs. If increased renal risk - Avoid NSAIDs	Str	1	.	.	.	.	.	.	.	.
40-22	Where paracetamol or topical NSAIDs provide insufficient pain relief for people with osteoarthritis, then the addition of or substitution with an oral NSAID/COX-2 inhibitor to paracetamol should be considered.	Cond	1	1	1	.	.	.	.	.	.
41-2	Topical or oral non-steroid anti-inflammatory drugs (NSAIDS) are recommended for the treatment of pain in patients waiting for surgery (LE 1a; LR A; LA 82%)	Str	1	1	.	.	.	.	.	.	.
5-13	Strong evidence supports that NSAIDs improve short-term pain, function, or both in patients with symptomatic osteoarthritis of the hip.	Str	.	1	.	.	.	.	.	.	.

QI v4_3: proportion using NSAIDs + PPI

Comment: 2 for; joints 2 knee; 1 for patients who failed SYSADOA/APAP/topical NSAIDs

id	verbatim_guideline_rec	LVL	Knee	Hip	Hand	Poly	Shldr	CV	GI	Frail	WPD
28-42	NSAIDs such as diclofenac, ibuprofen, and naproxen, and selective NSAIDs including celecoxib and etoricoxib are indicated in moderate pain. In all cases, gastric protection, such as a proton-pump inhibitor, is required and naproxen is recommended in patients with cardiovascular risk.	Str	1	.	.	.	.	.	.	.	.
4-16	As advanced pharmacological management in the persistent symptomatic patient (STEP 2) who did not respond to Step 1 background treatment, use intermittent or longer cycles of oral NSAIDs. If normal GI risk - use non-selective NSAID with PPI, or Cox-2 selective NSAID (preferred with concomitant PPI)	NS	1	.	.	.	.	.	1	.	.

QI v4_4: proportion using acetaminophen

Comment: 11 for & 5 against; joints 12 knee, 6 hip, 4 hand, 1 PA, 1 shoulder, 1 ankle

id	verbatim_guideline_rec	LVL	Knee	Hip	Hand	Poly	Shldr	CV	GI	Frail	WPD
1-50	Acetaminophen is conditionally recommended for patients with knee, hip, and/or hand OA.	Cond	1	1	1	.	.	.	.	.	.
12-19	A eight-week concentric-eccentric isokinetic or isometric exercise programs + paracetamol (cycling warm-up before the exercises; application of isokinetic dynamometer for exercises; cool-down after the exercises) (three days weekly) for the management of knee osteoarthritis for pain relief during rest (Visual Analogue Scale (VAS)), pain relief during motion (VAS), improved physical function (Western Ontario and McMaster Universities Arthritis Index (WOMAC) subscale) and improved quality of life (Short Form 36 (SF-36)) subscale) at eight-weeks end of treatment and at 12-weeks follow-up is recommended.	NS	1	.	.	.	.	.	.	.	.
2-102	Level 4B/4A conditional recommendation against Paracetamol	Cond	.	1	.	.	.	.	.	.	.
2-183	Level 4A conditional recommendation against Paracetamol	Cond	.	.	.	1	.	.	.	.	.
2-29	Level 4B/4A conditional recommendation against Paracetamol	Cond	1	.	.	.	.	.	.	.	.
22-8	We suggest offering acetaminophen and/or oral non-steroidal anti-inflammatory drugs for pain associated with osteoarthritis of the hip and knee.	Cond	1	1	.	.	.	.	.	.	.
28-29	The use of acetaminophen/paracetamol is recommended in mild to moderate pain, owing to its safety profile	Str	.	1	.	.	.	.	.	.	.
28-32	Acetaminophen/paracetamol is recommended at a dose of up to 3 g/day for the treatment of mild pain resulting from knee OA. Moderate gastrolesive effects may occur and patients should be monitored for possible hepatic complications.	Str	1	.	.	.	.	.	.	.	.
28-66	Acetaminophen/paracetamol (up to 3 g/day) is the preferred oral analgesic for the long-term treatment particularly in elderly patients because of its relative safety in comparison with NSAIDs.	Str	.	.	1	.	.	.	.	.	.
3-4	Paracetamol (acetaminophen) (up to 3 g/day) is an effective initial oral analgesic for treatment of mild to moderate pain. In elderly patients it should be preferred because of its relative safety in comparison with NSAIDs.	NS	1	1	1	.	.	.	.	.	.
30-1	Paracetamol must not necessarily be prescribed systematically and/or continuously (Level 1A, Strength A)	Str	1	.	.	.	.	.	.	.	.
30-4	Weak opioids, alone or in combination with paracetamol, may be proposed for analgesic purposes (Level 1A, Strength A) and must be prescribed while taken into account comorbidities and after providing information about adverse effects (Level 4, Strength D)	Str	1	.	.	.	.	.	.	.	.
31-4	Acetaminophen can be used in addition to primary precautions in patients who have mild disease with intermittent pain, short-term stiffness after inactivation and radiological knee OA of grade 0-1.	NS	1	.	.	.	.	.	.	.	.
4-2	A weak recommendation that paracetamol (acetaminophen) should not be used on a regular basis as Step 1 long-term background pharmacological therapy	Cond	1	.	.	.	.	.	.	.	.
4-5	A weak recommendation that paracetamol (acetaminophen) at doses no greater than 3 g/day may be used as short-term rescue analgesia only, given on top of a background of Step 1 chronic therapy with SYSADOAs	Cond	1	.	.	.	.	.	.	.	.
40-17	Healthcare professionals should consider offering paracetamol for pain relief in addition to core treatments; regular dosing may be required.	Cond	1	1	1	.	1	.	.	.	.
40-19	Consider topical NSAIDs for pain relief in addition to core treatments for people with knee or hand osteoarthritis. Consider topical NSAIDs and/or paracetamol ahead of oral NSAIDs, COX-2 inhibitors or opioids	NS	1	.	1	.	.	.	.	.	.

QI v4_5: proportion using bisphosphonates

Comment: 6 against; joints 3 knee, 3 hip, 2 hand, 1 poly

id	verbatim_guideline_rec	LVL	Knee	Hip	Hand	Poly	Shldr	CV	GI	Frail	WPD
1-27	Bisphosphonates are strongly recommended against in patients with knee, hip, and/or hand OA.	Str	1	1	1	.	.	.	.	.	.
2-126	Level 5 strong recommendation against Bisphosphonates	Str	.	1	.	.	.	.	.	.	.
2-201	Level 5 strong recommendation against Bisphosphonates	Str	.	.	.	1	.	.	.	.	.
2-59	Level 5 strong recommendation against Bisphosphonates	Str	1	.	.	.	.	.	.	.	.
28-26	Bisphosphonates (clodronate) is not recommended	Str	.	.	1	.	.	.	.	.	.
36-55	We suggest not offering bisphosphonates for people with knee and/or hip OA	Cond	1	1	.	.	.	.	.	.	.

QI v4_6: proportion using calcitonin

Comment: 4 against; joints 2 knee, 2 hip, 1 poly

id	verbatim_guideline_rec	LVL	Knee	Hip	Hand	Poly	Shldr	CV	GI	Frail	WPD
2-127	Level 5 strong recommendation against Calcitonin	Str	.	1	.	.	.	.	.	.	.
2-202	Level 5 strong recommendation against Calcitonin	Str	.	.	.	1	.	.	.	.	.
2-60	Level 5 strong recommendation against Calcitonin	Str	1	.	.	.	.	.	.	.	.
36-56	We suggest not offering calcitonin for people with knee and/or hip OA	Cond	1	1	.	.	.	.	.	.	.

QI v4_7: proportion using colchicine

Comment: 5 against; joints 3 knee, 3 hip, 1 hand, 1 poly

id	verbatim_guideline_rec	LVL	Knee	Hip	Hand	Poly	Shldr	CV	GI	Frail	WPD
1-55	Colchicine is conditionally recommended against in patients with knee, hip, and/or hand OA.	Cond	1	1	1	.	.	.	.	.	.
2-128	Level 5 strong recommendation against Colchicine	Str	.	1	.	.	.	.	.	.	.
2-203	Level 5 strong recommendation against Colchicine	Str	.	.	.	1	.	.	.	.	.
2-61	Level 5 strong recommendation against Colchicine	Str	1	.	.	.	.	.	.	.	.
36-58	We suggest not offering colchicine for people with knee and/or hip OA	Cond	1	1	.	.	.	.	.	.	.

QI v4_8: proportion using combinations of NSAIDs

Comment: 1 against; joints 1 knee

id	verbatim_guideline_rec	LVL	Knee	Hip	Hand	Poly	Shldr	CV	GI	Frail	WPD
8-3	The combination of NSAIDs is not recommended by the ESCEO task force	NS	1	.	.	.	.	.	.	.	.

QI v4_9: proportion using cox-2 inhibitors

Comment: 4 for (GI risk and widespread pain) & 7 against (frailty and CV risk); joints 5 knee, 5 hip, 2 hand, 3 poly

id	verbatim_guideline_rec	LVL	Knee	Hip	Hand	Poly	Shldr	CV	GI	Frail	WPD
2-10	Level 1B conditional recommendation for COX-2 Inhibitors	Cond	1	.	.	.	.	.	1	.	1
2-11	Level 3 conditional recommendation against COX-2 Inhibitors	Cond	1	.	.	.	.	.	.	1	.
2-12	Level 5 strong recommendation against COX-2 Inhibitors	Str	1	.	.	.	.	1	.	.	.
2-157	Level 1B/2 conditional recommendation for COX-2 Inhibitors	Cond	.	.	.	1	.	.	1	.	1
2-158	Level 3/4B conditional recommendation against COX-2 Inhibitors	Cond	.	.	.	1	.	.	.	1	.
2-159	Level 5 strong recommendation against COX-2 Inhibitors	Str	.	.	.	1	.	1	.	.	.
2-86	Level 1B/2 conditional recommendation for COX-2 Inhibitors	Cond	.	1	.	.	.	.	1	.	1
2-87	Level 3/4B conditional recommendation against COX-2 Inhibitors	Cond	.	1	.	.	.	.	.	1	.
2-88	Level 5 strong recommendation against COX-2 Inhibitors	Str	.	1	.	.	.	1	.	.	.
3-12	In patients with increased cardiovascular risk, naproxen can be used; COX-2 inhibitors are contraindicated and the other non-selective NSAIDs should be used with caution	Cond	1	1	1	.	.	1	.	.	.
3-26	In patients with increased gastrointestinal risk, non-selective NSAIDs plus a protonpump inhibitor, or a selective COX-2 inhibitor, should be used.	NS	1	1	1	.	.	.	1	.	.

QI v4_10: proportion using cox-2 inhibitors + PPI

Comment: 2 for if GI risk; joints 2 knee, 1 hip, 1 hand

id	verbatim_guideline_rec	LVL	Knee	Hip	Hand	Poly	Shldr	CV	GI	Frail	WPD
35-7	The combination of COX-2 inhibitor and proton pump inhibitor is recommended for patients with high risk of gastrointestinal adverse reactions (1B)	Str	1	1	1	.	.	.	1	.	.
4-28	Patients with persistent pain (STEP 2) need oral NSAIDS, if increased GI risk: Prefer Cox-2 selective NSAID (celecoxib) with PPI, Be mindful of complications with any NSAID	NS	1	.	.	.	.	.	1	.	.

QI v4_11: proportion using doxycycline

Comment: 4 against; joints 2 knee, 2 hip, 1 poly

id	verbatim_guideline_rec	LVL	Knee	Hip	Hand	Poly	Shldr	CV	GI	Frail	WPD
2-130	Level 5 strong recommendation against Doxycycline	Str	.	1	.	.	.	.	.	.	.
2-205	Level 5 strong recommendation against Doxycycline	Str	.	.	.	1	.	.	.	.	.
2-63	Level 5 strong recommendation against Doxycycline	Str	1	.	.	.	.	.	.	.	.
36-8	We do not recommend offering doxycycline for people with knee and/or hip OA	Str	1	1	.	.	.	.	.	.	.

QI v4_12: proportion using duloxetine

Comment: 10 for & 3 against; joints 11 knee, 5 hip, 2 hand, 1 poly

id	verbatim_guideline_rec	LVL	Knee	Hip	Hand	Poly	Shldr	CV	GI	Frail	WPD
1-54	Duloxetine is conditionally recommended for patients with knee, hip, and/or hand OA.	Cond	1	1	1	.	.	.	.	.	.
2-170	Level 3/4B/4A conditional recommendation against Duloxetine	Cond	.	.	.	1	.	.	.	.	.
2-20	Level 2 conditional recommendation for Duloxetine	Cond	1	.	.	.	.	.	.	.	1
2-21	Level 3/4A conditional recommendation against Duloxetine	Cond	1	.	.	.	.	1	1	1	.
2-98	Level 3/4A conditional recommendation against Duloxetine	Cond	.	1	.	.	.	.	.	.	.
22-9	We suggest offering duloxetine as an alternative or adjunctive therapy for patients with an inadequate response or contraindications to acetaminophen or non-steroidal anti-inflammatory drugs for pain associated with osteoarthritis of the knee.	Cond	1	.	.	.	.	.	.	.	.
28-19	Duloxetine may be helpful for knee OA accompanied by chronic pain	Cond	1	.	.	.	.	.	.	.	.
3-22	Duloxetine may be helpful for knee (and maybe hip) OA accompanied by chronic pain.	NS	1	1	.	.	.	.	.	.	.
30-5	Off-label duloxetine can be considered in the absence of therapeutic alternatives (Level 1A, Strength C)	Cond	1	.	.	.	.	.	.	.	.
31-16	Duloxetine can be used in patients with chronic pain which is unresponsive to other treatment options for patients with moderate-severe symptoms, functional capacity of either normal or minimally limited and/or radiologic grade of 2-3	NS	1	.	.	.	.	.	.	.	.
35-4	For OA patients with NSAIDs contraindications or ineffective pain treatment, it is suggested to take opioids or duloxetine for analgesia (2C)	NS	1	1	1	.	.	.	.	.	.
36-26	It may be appropriate to offer duloxetine for some people with knee and/or hip OA	Cond	1	1	.	.	.	.	.	.	.
4-24	A weak recommendation to the use of duloxetine as an alternative to weak opioids in Step 3 of the algorithm, especially in patients with pain from central sensitization.	Cond	1	.	.	.	.	.	.	.	.

QI v4_13: proportion using hydroxychloroquine

Comment: 2 against; joints 1 knee, 1 hip, 2 hand

id	verbatim_guideline_rec	LVL	Knee	Hip	Hand	Poly	Shldr	CV	GI	Frail	WPD
1-48	Hydroxychloroquine is strongly recommended against in patients with knee, hip, and/or hand OA.	Str	1	1	1	.	.	.	.	.	.
28-37	Hydroxychloroquine is not recommended for the symptomatic treatment of erosive hand OA	Str	.	.	1	.	.	.	.	.	.

QI v4_14: proportion using methotrexate

Comment: 5 against; joints 3 knee, 3 hip, 1 hand, 1 poly

id	verbatim_guideline_rec	LVL	Knee	Hip	Hand	Poly	Shldr	CV	GI	Frail	WPD
1-9	Methotrexate is strongly recommended against in patients with knee, hip, and/or hand OA.	Str	1	1	1	.	.	.	.	.	.
2-139	Level 5 strong recommendation against Methotrexate	Str	.	1	.	.	.	.	.	.	.
2-213	Level 5 strong recommendation against Methotrexate	Str	.	.	.	1	.	.	.	.	.
2-69	Level 5 strong recommendation against Methotrexate	Str	1	.	.	.	.	.	.	.	.
36-59	We suggest not offering methotrexate for people with knee and/or hip OA	Cond	1	1	.	.	.	.	.	.	.

QI v4_15: proportion using naproxen

Comment: 1 for if CV risk; joints 1 knee, 1 hip, 1 hand

id	verbatim_guideline_rec	LVL	Knee	Hip	Hand	Poly	Shldr	CV	GI	Frail	WPD
3-12	In patients with increased cardiovascular risk, naproxen can be used; COX-2 inhibitors are contraindicated and the other non-selective NSAIDs should be used with caution	Cond	1	1	1	.	.	1	.	.	.

QI v4_16: proportion using naproxen + PPI

Comment: 2 for if CV + GI risk; joints 1 knee, 1 hip

id	verbatim_guideline_rec	LVL	Knee	Hip	Hand	Poly	Shldr	CV	GI	Frail	WPD
28-42	NSAIDs such as diclofenac, ibuprofen, and naproxen, and selective NSAIDs including celecoxib and etoricoxib are indicated in moderate pain. In all cases, gastric protection, such as a proton-pump inhibitor, is required and naproxen is recommended in patients with cardiovascular risk.	Str	1	.	.	.	.	1	.	.	.
28-55	Naproxen could be used in patients with cardiovascular risk. It should be administered in conjunction with a proton-pump inhibitor owing to the high gastrointestinal risk	Str	.	1	.	.	.	1	1	.	.

QI v4_17: proportion using non-selective NSAIDs

Comment: 3 for (widespread pain) & 3 against (CV/GI); joints 2 knee, 2 hip, 2 poly

id	verbatim_guideline_rec	LVL	Knee	Hip	Hand	Poly	Shldr	CV	GI	Frail	WPD
2-153	Level 1B/2 conditional recommendation for Non-selective NSAIDs	Cond	.	.	.	1	.	.	.	.	1
2-154	Level 3/4B/4A conditional recommendation against Non-selective NSAIDs	Cond	.	.	.	1	.	1	1	1	.
2-6	Level 1B conditional recommendation for Non-selective NSAIDs	Cond	1	.	.	.	.	.	.	.	1
2-7	Level 3/4B conditional recommendation against Non-selective NSAIDs	Cond	1	.	.	.	.	1	.	1	.
2-82	Level 1B/2 conditional recommendation for Non-selective NSAIDs	Cond	.	1	.	.	.	.	.	.	1
2-83	Level 3/4B/4A conditional recommendation against Non-selective NSAID	Cond	.	1	.	.	.	1	1	1	.

QI v4_18: proportion using non-selective NSAIDs + PPI

Comment: 4 for (GI/widespread pain) & 3 against (frail/CV risk); joints 3 knee, 3 hip, 1 hand, 2 poly

id	verbatim_guideline_rec	LVL	Knee	Hip	Hand	Poly	Shldr	CV	GI	Frail	WPD
2-155	Level 2 conditional recommendation for Non-selective NSAID+PPI	Cond	.	.	.	1	.	.	1	.	1
2-156	Level 3/4B conditional recommendation against Non-selective NSAID+PPI	Cond	.	.	.	1	.	1	.	1	.
2-8	Level 1B/2 conditional recommendation for Non-selective NSAIDs + PPI	Cond	1	.	.	.	.	.	1	.	1
2-84	Level 2 conditional recommendation for Non-selective NSAID + PPI	Cond	.	1	.	.	.	.	1	.	1
2-85	Level 3/4B conditional recommendation against Non-selective NSAID + PPI	Cond	.	1	.	.	.	1	.	1	.
2-9	Level 3/4B conditional recommendation against Non-selective NSAIDs + PPI	Cond	1	.	.	.	.	1	.	1	.
3-26	In patients with increased gastrointestinal risk, non-selective NSAIDs plus a protonpump inhibitor, or a selective COX-2 inhibitor, should be used.	NS	1	1	1	.	.	.	1	.	.

QI v4_19: proportion using opioids

Comment: 11 for & 17 against; joints 18 knee, 13 hip, 4 hand, 5 poly, 2 shoulder; recommended as last pharmacological option for 7 if surgery not possible

id	verbatim_guideline_rec	LVL	Knee	Hip	Hand	Poly	Shldr	CV	GI	Frail	WPD
1-43	Non-tramadol opioids are conditionally recommended against in patients with knee, hand, and/or hip OA with the recognition that they may be used under certain circumstances, particularly when alternatives have been exhausted.	Cond	1	1	1	.	.	.	.	.	.
2-106	Level 4A conditional recommendation against Oral Opioids	Cond	.	1	.	.	.	.	.	.	.
2-107	Level 5 strong recommendation against Oral Opioids	Str	.	1	.	.	.	1	1	1	1
2-108	Level 5 strong recommendation against Oral Opioids strongly not recommended over NSAIDs	Str	.	1	.	.	.	.	.	.	.
2-149	Level 5 strong recommendation against Transdermal Opioids	Str	.	1	.	.	.	.	.	.	.
2-179	Level 4A conditional recommendation against Oral Opioids	Cond	.	.	.	1	.	.	.	.	.
2-180	Level 5 strong recommendation against Oral Opioids	Str	.	.	.	1	.	1	1	1	1
2-181	Level 5 strong recommendation against Oral Opioids over NSAIDs	Str	.	.	.	1	.	.	.	.	.
2-182	Level 5 strong recommendation against Transdermal Opioids	Str	.	.	.	1	.	.	.	.	.
2-34	Level 4A conditional recommendation against Oral Opioids	Cond	1	.	.	.	.	.	.	.	.
2-35	Level 5 strong recommendation against Oral Opioids	Str	1	.	.	.	.	1	1	1	1
2-36	Level 5 strong recommendation against Oral Opioids over NSAIDs	Str	1	.	.	.	.	.	.	.	.
2-79	Level 5 strong recommendation against Transdermal Opioids	Str	1	.	.	.	.	.	.	.	.
22-10	We suggest against initiating opioids (including tramadol) for pain associated with osteoarthritis of the hip and knee.	Cond	1	1	.	.	.	.	.	.	.
28-17	Weak opioids such as tramadol may be beneficial if there is no response to NSAIDs or COX-2 inhibitors, no toleration, or are contraindicated	Cond	.	1	.	.	.	.	.	.	.
3-21	The use of weak opioids in case of severe pain or no response, intolerance or contraindication to NSAIDs, is recommended. Stronger opioids should only be used for the management of severe pain in exceptional circumstances.	NS	1	1	1	.	.	.	.	.	.
30-13	Strong opioids must only be prescribed to patients who have a contraindication to knee surgery, who have failed or have a contraindication to other treatments, while taking into account comorbidities and after providing information about adverse effects (Level 4, Strength D)	Cond	1	.	.	.	.	.	.	.	.
30-4	Weak opioids, alone or in combination with paracetamol, may be proposed for analgesic purposes (Level 1A, Strength A) and must be prescribed while taken into account comorbidities and after providing information about adverse effects (Level 4, Strength D)	Str	1	.	.	.	.	.	.	.	.
31-13	Weak opioid analgesics can be given for a short period of time to the patients who are not responding to other options for patients who are severely symptomatic, and with limited functional capacity, deformities and/or radiological grade of 4	NS	1	.	.	.	.	.	.	.	.
34-4	In the absence of reliable evidence, it is the opinion of the work group that opioids not be prescribed because routine and long-term pain management of glenohumeral osteoarthritis	Cond	.	.	.	.	1	.	.	.	.
35-4	For OA patients with NSAIDs contraindications or ineffective pain treatment, it is suggested to take opioids or duloxetine for analgesia (2C)	NS	1	1	1	.	.	.	.	.	.

36-6	We do not recommend offering oral opioids for people with knee and/or hip OA	Str	1	1	.	.	.	.	.	.	.
36-7	We do not recommend offering transdermal opioids for people with knee and/or hip OA	Str	1	1	.	.	.	.	.	.	.
39-1	Opioids should only be used for chronic OA pain in these situations: Failure of non-pharmacological treatments; failure of or contraindication for other analgesics; joint replacement not possible or refused by the patient. Strong consensus-based recommendation, strong consensus.	Str	1	1	1	1	1	.	.	.	.
4-12	Last pharmacological options for the severely symptomatic patient are represented by short-term weak opioids, such as tramadol	Cond	1	.	.	.	.	.	.	.	.
4-22	A weak recommendation to the use of short-term weak opioids in Step 3 of the treatment algorithm as the last pharmacological attempt before surgery.	Cond	1	.	.	.	.	.	.	.	.
4-6	A weak recommendation to the use of classical oral or transdermal opioids in end-stage knee OA patients for whom surgery is contraindicated.	Cond	1	.	.	.	.	.	.	.	.
41-6	In patients with high levels of pain the use of strong opiates is recommended (LE 3a; LR C; LA 88%).	Cond	1	1	.	.	.	.	.	.	.

QI v4_20: proportion using oral steroids

Comment: 1 for in elderly; joints 1 knee

id	verbatim_guideline_rec	LVL	Knee	Hip	Hand	Poly	Shldr	CV	GI	Frail	WPD
28-11	The administration of low-dose oral steroids for a maximum of 12 weeks could be considered in patients older than 65 years	Cond	1	.	.	.	.	.	.	.	.

QI v4_21: proportion using pregabalin and gabapentin

Comment: 1 against; joints 1 hip, 1 knee

id	verbatim_guideline_rec	LVL	Knee	Hip	Hand	Poly	Shldr	CV	GI	Frail	WPD
41-8	The use of anti-epileptic drugs is advised against for these patients (LE 1b; LR A; LA 82%)	Str	1	1	.	.	.	.	.	.	.

QI v4_22: proportion using statins

Comment: 3 against; joints 1 hip, 1 knee, 1 poly

id	verbatim_guideline_rec	LVL	Knee	Hip	Hand	Poly	Shldr	CV	GI	Frail	WPD
2-144	Level 5 strong recommendation against Statins	Str	.	1	.	.	.	.	.	.	.
2-218	Level 5 strong recommendation against Statins	Str	.	.	.	1	.	.	.	.	.
2-76	Level 5 strong recommendation against Statins	Str	1	.	.	.	.	.	.	.	.

QI v4_23: proportion using sustained-release tramadol

Comment: 2 for; joints 2 knee

id	verbatim_guideline_rec	LVL	Knee	Hip	Hand	Poly	Shldr	CV	GI	Frail	WPD
4-18	If tramadol is selected as step 3 last pharmacological option for the severely symptomatic patient, use sustained-release (SR) formulation of tramadol maintaining upwards titration from 50 mg to 100 mg to improve tolerability and minimize treatment discontinuations due to AEs.	NS	1	.	.	.	.	.	.	.	.
8-4	The sustained-release (SR) formulation of tramadol is preferred and the slow upwards titration of tramadol SR is recommended to improve tolerability and minimize AE-related treatment discontinuations.	NS	1	.	.	.	.	.	.	.	.

QI v4_24: proportion using synthetic DMARDs

Comment: 2 against; joints 2 hand

id	verbatim_guideline_rec	LVL	Knee	Hip	Hand	Poly	Shldr	CV	GI	Frail	WPD
18-8	Conventional synthetic or biologic DMARDs should not be used in patients with hand OA.	NS	.	.	1	.	.	.	.	.	.
24-5	Patients with hand OA should not be treated with conventional or biological disease-modifying antirheumatic drugs	NS	.	.	1	.	.	.	.	.	.

QI v4_25: proportion using tramadol

Comment: 3 for; joints 3 knee, 1 hip, 1 hand

id	verbatim_guideline_rec	LVL	Knee	Hip	Hand	Poly	Shldr	CV	GI	Frail	WPD
1-12	Tramadol is conditionally recommended for patients with knee, hip, and/or OA.	Cond	1	1	1	.	.	.	.	.	.
28-14	The use of tramadol in the case of severe pain in its various administration forms is recommended	Str	1	.	.	.	.	.	.	.	1
8-15	The short-term use of tramadol may be considered for severely symptomatic OA patients and there is good evidence that tramadol works if prescribed properly	NS	1	.	.	.	.	.	.	.	.

Domain: 05 Support devices

No QIs were considered feasible for this domain

Domain: 06 Supplement

QI v6_1: proportion using chondroitin

Comment: 7 for & 8 against; joints 7 knee, 6 hip, 5 hand, 2 poly, 1 shoulder, 1 ankle

id	verbatim_guideline_rec	LVL	Knee	Hip	Hand	Poly	Shldr	CV	GI	Frail	WPD
1-14	Chondroitin sulfate is strongly recommended against in patients with knee and/or hip OA as are combination products that include glucosamine and chondroitin sulfate, but is conditionally recommended for patients with hand OA.	Str	1	1	.	.	.	.	.	.	.
1-22	Chondroitin sulfate is strongly recommended against in patients with knee and/or hip OA as are combination products that include glucosamine and chondroitin sulfate, but is conditionally recommended for patients with hand OA.	Cond	.	.	1	.	.	.	.	.	.
18-10	In patients who do not attain adequate benefit from paracetamol or topical/systemic NSAIDs or whom these agents are contraindicated, chondroitin sulfate may be used to reduce pain and improve functions.	NS	.	.	1	.	.	.	.	.	.
2-122	Level 4A conditional recommendation against Chondroitin	Cond	.	1	.	.	.	1	1	.	1
2-123	Level 5 strong recommendation against Chondroitin	Str	.	1	.	.	.	.	.	1	.
2-199	Level 4A conditional recommendation against Chondroitin	Cond	.	.	.	1	.	1	1	.	1
2-200	Level 5 strong recommendation against Chondroitin	Str	.	.	.	1	.	.	.	1	.
2-32	Level 4B/4A conditional recommendation against Chondroitin	Cond	1	.	.	.	.	.	.	.	.
24-10	Chondroitin sulfate may be used in patients with hand OA for pain relief and improvement in functioning	NS	.	.	1	.	.	.	.	.	.
28-49	Chondroitin sulfate has shown to have a beneficial effect on symptoms in patients with knee OA and a high safety profile. It has been proven that its effect persists for 3 months after stopping the treatment (carryover effect). Recent studies have provided evidence that chondroitin sulfate use may delay OA progression.	Str	1	.	.	.	.	.	.	.	.
28-61	The use of chondroitin sulfate for pain relief and function is recommended as it has a good safety profile	Str	.	.	1	.	.	.	.	.	.
31-14	Although the evidence about the use of glucosamine and/or chondroitin sulphate is uncertain, these agents may be used in patients who have mild disease with intermittent pain, short-term stiffness after inactivation and radiological knee OA of grade 0-1.	NS	1	.	.	.	.	.	.	.	.
35-11	For some patients, treatment with glucosamine or chondroitin sulfate can be selected. It should be stopped if no symptom improved after 3 to 6 months (2C)	NS	1	1	.	.	.	.	.	.	.
36-64	We suggest not offering chondroitin for people with knee and/or hip OA	Cond	1	1	.	.	.	.	.	.	.
40-11	Do not offer glucosamine or chondroitin products for the management of osteoarthritis	Str	1	1	1	.	1	.	.	.	.

QI v6_2: proportion using chondroitin + glucosamine

Comment: 4 for & 5 against; joints 7 knee, 2 hip, 1 hand, 1 poly

id	verbatim_guideline_rec	LVL	Knee	Hip	Hand	Poly	Shldr	CV	GI	Frail	WPD
2-135	Level 5 strong recommendation against Glucosamine & Chondroitin	Str	.	1	.	.	.	.	.	.	.
2-197	Level 4A conditional recommendation against Glucosamine + Chondroitin	Cond	.	.	.	1	.	.	.	.	.
2-57	Level 4A conditional recommendation against Glucosamine + Chondroitin	Cond	1	.	.	.	.	.	.	.	.
2-58	Level 5 strong recommendation against Glucosamine + Chondroitin	Str	1	.	.	.	.	1	1	1	1
28-65	The combined use of glucosamine and chondroitin sulfate is indicated in patients with knee OA and moderate to severe pain	Str	1	.	.	.	.	.	.	.	.
28-7	Glucosamine and chondroitin sulfate are supported in the treatment of hand and knee OA	Str	1	.	1	.	.	.	.	.	.
3-25	In patients with symptomatic knee OA, glucosamine sulphate and chondroitin sulphate may have beneficial effect on symptoms. Structural effects, patients suitable for treatment and the cost to benefit ratio of the therapy remain to be defined.	NS	1	.	.	.	.	.	.	.	.
31-14	Although the evidence about the use of glucosamine and/or chondroitin sulphate is uncertain, these agents may be used in patients who have mild disease with intermittent pain, short-term stiffness after inactivation and radiological knee OA of grade 0-1.	NS	1	.	.	.	.	.	.	.	.
41-3	The use of glucosamine and chondroitin sulfate is advised against for these patients (LE 1b; LR A; LA 82%)	Str	1	1	.	.	.	.	.	.	.

QI v6_3: proportion using collagen

Comment: 4 against; joints 1 hip, 2 knee, 1 poly

id	verbatim_guideline_rec	LVL	Knee	Hip	Hand	Poly	Shldr	CV	GI	Frail	WPD
2-121	Level 4A conditional recommendation against Collagen	Cond	.	1	.	.	.	.	.	.	.
2-194	Level 4A conditional recommendation against Collagen	Cond	.	.	.	1	.	.	.	.	.
2-54	Level 4A conditional recommendation against Collagen	Cond	1	.	.	.	.	1	1	.	1
2-55	Level 5 strong recommendation against Collagen	Str	1	.	.	.	.	.	.	1	.

QI v6_4: proportion using combination formulation of chondroitin and glucosamine

Comment: 3 against; joints 3 knee, 2 hip

id	verbatim_guideline_rec	LVL	Knee	Hip	Hand	Poly	Shldr	CV	GI	Frail	WPD
1-14	Chondroitin sulfate is strongly recommended against in patients with knee and/or hip OA as are combination products that include glucosamine and chondroitin sulfate, but is conditionally recommended for patients with hand OA.	Str	1	1	.	.	.	.	.	.	.
36-65	We suggest not offering glucosamine and chondroitin in compound form for people with knee and/or hip OA	Cond	1	1	.	.	.	.	.	.	.
4-15	A weak recommendation that a combination of glucosamine and CS should not be used in Step 1 of background therapy, as there is no preparation containing both prescription products and no convincing evidence for existing non-prescription formulations.	Cond	1	.	.	.	.	.	.	.	.

QI v6_5: proportion using glucosamine

Comment: 3 for & 7 against; joints 7 knee, 6 hip, 2 hand, 1 poly, 1 shoulder, 1 ankle

id	verbatim_guideline_rec	LVL	Knee	Hip	Hand	Poly	Shldr	CV	GI	Frail	WPD
1-51	Glucosamine is strongly recommended against in patients with knee, hip, and/or hand OA.	Str	1	1	1	.	.	.	.	.	.
2-134	Level 5 strong recommendation against Glucosamine	Str	.	1	.	.	.	.	.	.	.
2-208	Level 5 strong recommendation against Glucosamine	Str	.	.	.	1	.	.	.	.	.
2-65	Level 5 strong recommendation against Glucosamine	Str	1	.	.	.	.	.	.	.	.
28-63	Glucosamine may be beneficial for pain relief and for improving joint function in patients	Str	1	.	.	.	.	.	.	.	.
31-14	Although the evidence about the use of glucosamine and/or chondroitin sulphate is uncertain, these agents may be used in patients who have mild disease with intermittent pain, short-term stiffness after inactivation and radiological knee OA of grade 0-1.	NS	1	.	.	.	.	.	.	.	.
35-11	For some patients, treatment with glucosamine or chondroitin sulfate can be selected. It should be stopped if no symptom improved after 3 to 6 months (2C)	NS	1	1	.	.	.	.	.	.	.
36-63	We suggest not offering glucosamine for people with knee and/or hip OA	Cond	1	1	.	.	.	.	.	.	.
40-11	Do not offer glucosamine or chondroitin products for the management of osteoarthritis	Str	1	1	1	.	1	.	.	.	.
5-2	Moderate strength evidence does not support the use of glucosamine sulfate because it did not perform better than placebo for improving function, reducing stiffness and decreasing pain for patients with symptomatic osteoarthritis of the hip.	NS	.	1	.	.	.	.	.	.	.

QI v6_6: proportion using methylsulfonylmethane

Comment: 3 against; joints 1 hip, 1 knee, 1 poly

id	verbatim_guideline_rec	LVL	Knee	Hip	Hand	Poly	Shldr	CV	GI	Frail	WPD
2-104	Level 4B/4A conditional recommendation against Methylsulfonlymethane	Cond	.	1	.	.	.	.	.	.	.
2-198	Level 4A conditional recommendation against Methylsulfonylmethane	Cond	.	.	.	1	.	.	.	.	.
2-31	Level 4B/4A conditional recommendation against Methylsulfonylmethane	Cond	1	.	.	.	.	.	.	.	.

QI v6_7: proportion using omega 3/6 fatty acids

Comment: 5 against; joints 3 hip, 3 knee, 1 hand, 1 poly

id	verbatim_guideline_rec	LVL	Knee	Hip	Hand	Poly	Shldr	CV	GI	Frail	WPD
1-1	Fish oil is conditionally recommended against in patients with knee, hip, and/or hand OA.	Cond	1	1	1	.	.	.	.	.	.
2-141	Level 5 strong recommendation against (Omega-3/6) Poly-Unsaturated Fatty Acids	Str	.	1	.	.	.	.	.	.	.
2-215	Level 5 strong recommendation against (Omega-3/6) Poly-Unsaturated Fatty Acids	Str	.	.	.	1	.	.	.	.	.
2-70	Level 5 strong recommendation against (Omega-3/6) Poly-Unsaturated Fatty Acids	Str	1	.	.	.	.	.	.	.	.
36-67	We suggest not offering omega-3 fatty acids for people with knee and/or hip OA	Cond	1	1	.	.	.	.	.	.	.

QI v6_8: proportion using oral hyaluronic acid

Comment: 1 for; joints 1 knee

id	verbatim_guideline_rec	LVL	Knee	Hip	Hand	Poly	Shldr	CV	GI	Frail	WPD
28-23	Oral administration of hyaluronic acid may have a beneficial therapeutic effect in patients with symptomatic knee OA and may possibly have an even greater effect in relatively young patients	Cond	1	.	.	.	.	.	.	.	.

QI v6_9: proportion using prescription chondroitin sulfate

Comment: 3 for; joints 3 knee

id	verbatim_guideline_rec	LVL	Knee	Hip	Hand	Poly	Shldr	CV	GI	Frail	WPD
4-7	A strong recommendation to the use of prescription CS (chondroitin sulfate) as Step 1 long-term background therapy, as an alternative to pCGS, and the prescription drug should be distinguished from low quality OTC products.	Str	1	.	.	.	.	.	.	.	.
8-2	For SYSADOAs (symptomatic slow-acting drugs for osteoarthritis), a high-quality evidence is provided only for the prescription formulations of patented crystalline glucosamine sulfate (pcGS) and chondroitin sulfate (CS)	NS	1	.	.	.	.	.	.	.	.
8-7	During step1, background treatment with SYSADOAs using only the prescription formulations of patented crystalline glucosamine sulfate (pCGS) or chondroitin sulfate (CS) is recommended, with paracetamol as add-on rescue analgesia for short-term therapy.	NS	1	.	.	.	.	.	.	.	.

QI v6_10: proportion using vitamin D

Comment: 5 against; joints 3 hip, 3 knee, 1 hand, 1 poly

id	verbatim_guideline_rec	LVL	Knee	Hip	Hand	Poly	Shldr	CV	GI	Frail	WPD
1-17	Vitamin D is conditionally recommended against in patients with knee, hip, and/or hand OA.	Cond	1	1	1	.	.	.	.	.	.
2-105	Level 4B/4A conditional recommendation against Vitamin D	Cond	.	1	.	.	.	.	.	.	.
2-178	Level 4B/4A conditional recommendation against Vitamin D	Cond	.	.	.	1	.	.	.	.	.
2-33	Level 4B/4A conditional recommendation against Vitamin D	Cond	1	.	.	.	.	.	.	.	.
36-66	We suggest not offering vitamin D for people with knee and/or hip OA	Cond	1	1	.	.	.	.	.	.	.

Domain: 07 Alternative therapy

QI v7_1: proportion using TENS

Comment: 5 for; joints 4 knee, 4 hip, 1 hand, 1 shoulder, 1 ankle

id	verbatim_guideline_rec	LVL	Knee	Hip	Hand	Poly	Shldr	CV	GI	Frail	WPD
28-60	Transcutaneous electrical nerve stimulation (TENS) should also be used for pain relief and to reduce stiffness	Cond	.	1	.	.	.	.	.	.	.
3-30	Transcutaneous electrical nerve stimulation (TENS) may help with short-term pain control in some patients with hip or knee OA	NS	1	1	.	.	.	.	.	.	.
31-7	The electrotherapeutic agents with analgesic properties (TENS, interferential and diadynamic current) may have beneficial effects over pain, functional status and quality of life and can be used at all stages of the disease.	NS	1	.	.	.	.	.	.	.	.
36-25	It may be appropriate to offer TENS that can be used at home for some people with knee and/or hip OA	Cond	1	1	.	.	.	.	.	.	.
40-10	Healthcare professionals should consider the use of transcutaneous electrical nerve stimulation (TENS) as an adjunct to core treatments for pain relief.	Cond	1	1	1	.	1	.	.	.	.

QI v7_2: proportion using acupuncture

Comment: 5 for & 12 against; joints 11 knee, 7 hip, 2 hand, 3 poly, 1 shoulder, 1 ankle

id	verbatim_guideline_rec	LVL	Knee	Hip	Hand	Poly	Shldr	CV	GI	Frail	WPD
1-40	Acupuncture is conditionally recommended for patients with knee, hip, and/or hand OA.	Cond	1	1	1	.	.	.	.	.	.
2-111	Level 4A conditional recommendation against Conventional Acupuncture	Cond	.	1	.	.	.	.	.	.	.
2-112	Level 5 strong recommendation against Laser Acupuncture	Str	.	1	.	.	.	.	.	.	.
2-132	Level 5 strong recommendation against Electroacupuncture	Str	.	1	.	.	.	.	.	.	.
2-185	Level 4A conditional recommendation against Conventional Acupuncture	Cond	.	.	.	1	.	.	.	.	.
2-206	Level 5 strong recommendation against Electroacupuncture	Str	.	.	.	1	.	.	.	.	.
2-211	Level 5 strong recommendation against Laser Acupuncture	Str	.	.	.	1	.	.	.	.	.
2-37	Level 4A conditional recommendation against Conventional Acupuncture	Cond	1	.	.	.	.	.	.	.	.
2-38	Level 4A conditional recommendation against Electroacupuncture	Cond	1	.	.	.	.	.	.	.	1
2-39	Level 5 strong recommendation against Electroacupuncture	Str	1	.	.	.	.	1	1	1	.
2-40	Level 5 strong recommendation against Laser Acupuncture	Str	1	.	.	.	.	.	.	.	.
20-2	Physical passive interventions of manual therapy, ultrasound, acupuncture (high dose at least 3 times per week), and low-level laser therapy are recommended, based on current evidence, for knee OA.	NS	1	.	.	.	.	.	.	.	.
35-1	Physical therapy such as manipulation therapy, massage, and acupuncture is recommended for OA patients to relieve pain and improve physical function (2B)	NS	1	1	.	.	.	.	.	.	.
36-53	We suggest not offering acupuncture (ie traditional, laser, electrical) for people with knee and/or hip OA	Cond	1	1	.	.	.	.	.	.	.
40-12	Do not offer acupuncture for the management of osteoarthritis	Str	1	1	1	.	1	.	.	.	.
6-3	Nonpharmacologic Interventions: Acupotomology - Positions including suprapatellar bursa, infrapatellar fat pad, Neixiyan, Waixiyan, tibia collateral ligament, iliotibial band, pes anserinus bursa and other parts around the knee can be beneficial.	NS	1	.	.	.	.	.	.	.	.
6-8	Nonpharmacologic Interventions: The acupuncture treatments should be utilized in combination of local point and acupoints along channel.	NS	1	.	.	.	.	.	.	.	.

QI v7_3: proportion using electrical stimulation (TENS, inferential, neuromuscular ENS, or pulsed/non-pulsed muscle stimulation)

Comment: 7 against; joints 4 knee, 4 hip, 2 poly

id	verbatim_guideline_rec	LVL	Knee	Hip	Hand	Poly	Shldr	CV	GI	Frail	WPD
1-35	Transcutaneous electrical stimulation (TENS) is strongly recommended against in patients with knee and/or hip OA.	Str	1	1	.	.	.	.	.	.	.
2-131	Level 5 strong recommendation against Electrical Stimulation	Str	.	1	.	.	.	.	.	.	.
2-192	Level 4A conditional recommendation against Electrical Stimulation	Cond	.	.	.	1	.	1	1	.	.
2-193	Level 5 strong recommendation against Electrical Stimulation	Str	.	.	.	1	.	.	.	1	1
2-64	Level 5 strong recommendation against Electrical Stimulation	Str	1	.	.	.	.	.	.	.	.
36-51	We suggest not offering electrotherapy modalities of shockwave, interferential or laser for people with knee and/or hip OA	Cond	1	1	.	.	.	.	.	.	.
38-7	Preferably do not offer treatment with TENS therapy to patients with hip or knee OA. Consider the use of TENS only as a brief intervention for pain reduction to support exercise therapy if exercise therapy is being hampered due to severe pain symptoms.	Cond	1	1	.	.	.	.	.	.	.

QI v7_4: proportion using electromagnetic therapy

Comment: 4 against; joints 2 knee, 2 hip, 1 poly

id	verbatim_guideline_rec	LVL	Knee	Hip	Hand	Poly	Shldr	CV	GI	Frail	WPD
2-133	Level 5 strong recommendation against Electromagnetic Therapy	Str	.	1	.	.	.	.	.	.	.
2-207	Level 5 strong recommendation against Electromagnetic Therapy	Str	.	.	.	1	.	.	.	.	.
2-41	Level 4A conditional recommendation against Electromagnetic Therapies	Cond	1	.	.	.	.	.	.	.	.
38-8	Do not offer continuous passive motion (CPM) (after total joint replacement surgery), pulsed electromagnetic field therapy, low-level laser therapy (LLLT), passive mobilizations, shock wave therapy, taping, thermotherapy, or ultrasound therapy to patients with hip or knee OA.	Str	1	1	.	.	.	.	.	.	.

QI v7_5: proportion using iontophoresis

Comment: 1 against; joints 1 hand

id	verbatim_guideline_rec	LVL	Knee	Hip	Hand	Poly	Shldr	CV	GI	Frail	WPD
1-19	Iontophoresis is conditionally recommended against in patients with first carpometacarpal (CMC) joint OA.	Cond	.	.	1	.	.	.	.	.	.

QI v7_6: proportion using kinesio taping

Comment: 1 for & 4 against; joints 5 knee, 2 hip, 1 hand

id	verbatim_guideline_rec	LVL	Knee	Hip	Hand	Poly	Shldr	CV	GI	Frail	WPD
1-25	Kinesiotaping is conditionally recommended for patients with knee and/or first CMC joint OA.	Cond	1	.	1	.	.	.	.	.	.
2-68	Level 5 strong recommendation against Kinesio Taping/Strapping	Str	1	.	.	.	.	.	.	.	.
2-72	Level 5 strong recommendation against Patellar Taping	Str	1	.	.	.	.	.	.	.	.
36-50	We suggest not offering kinesio taping for people with knee and/or hip OA	Cond	1	1	.	.	.	.	.	.	.
38-8	Do not offer continuous passive motion (CPM) (after total joint replacement surgery), pulsed electromagnetic field therapy, low-level laser therapy (LLLT), passive mobilizations, shock wave therapy, taping, thermotherapy, or ultrasound therapy to patients with hip or knee OA.	Str	1	1	.	.	.	.	.	.	.

QI v7_7: proportion using laser therapy

Comment: 1 for & 5 against; joints 4 knee, 3 hip, 1 poly

id	verbatim_guideline_rec	LVL	Knee	Hip	Hand	Poly	Shldr	CV	GI	Frail	WPD
2-138	Level 5 strong recommendation against Laser Therapy	Str	.	1	.	.	.	.	.	.	.
2-212	Level 5 strong recommendation against Laser Therapy	Str	.	.	.	1	.	.	.	.	.
2-24	Level 3/4B/4A conditional recommendation against Laser Therapy	Cond	1	.	.	.	.	.	.	.	.
20-2	Physical passive interventions of manual therapy, ultrasound, acupuncture (high dose at least 3 times per week), and low-level laser therapy are recommended, based on current evidence, for knee OA.	NS	1	.	.	.	.	.	.	.	.
36-51	We suggest not offering electrotherapy modalities of shockwave, interferential or laser for people with knee and/or hip OA	Cond	1	1	.	.	.	.	.	.	.
38-8	Do not offer continuous passive motion (CPM) (after total joint replacement surgery), pulsed electromagnetic field therapy, low-level laser therapy (LLLT), passive mobilizations, shock wave therapy, taping, thermotherapy, or ultrasound therapy to patients with hip or knee OA.	Str	1	1	.	.	.	.	.	.	.

QI v7_8: proportion using nerve block therapy

Comment: 3 against; joints 1 knee, 1 hip, 1 poly

id	verbatim_guideline_rec	LVL	Knee	Hip	Hand	Poly	Shldr	CV	GI	Frail	WPD
2-140	Level 5 strong recommendation against Nerve Block Therapy	Str	.	1	.	.	.	.	.	.	.
2-214	Level 5 strong recommendation against Nerve Block Therapy	Str	.	.	.	1	.	.	.	.	.
2-43	Level 4A conditional recommendation against Nerve Block Therapy	Cond	1	.	.	.	.	.	.	.	.

QI v7_9: proportion using pulsed vibration therapy

Comment: 1 against; joints 1 knee

id	verbatim_guideline_rec	LVL	Knee	Hip	Hand	Poly	Shldr	CV	GI	Frail	WPD
1-47	Pulsed vibration therapy is conditionally recommended against in patients with knee OA.	Cond	1	.	.	.	.	.	.	.	.

QI v7_10: proportion using radiofrequency (shockwave or ablation)

Comment: 1 for & 2 against; joints 3 knee, 2 hip

id	verbatim_guideline_rec	LVL	Knee	Hip	Hand	Poly	Shldr	CV	GI	Frail	WPD
1-46	Radiofrequency ablation is conditionally recommended for patients with knee OA.	Cond	1	.	.	.	.	.	.	.	.
36-51	We suggest not offering electrotherapy modalities of shockwave, interferential or laser for people with knee and/or hip OA	Cond	1	1	.	.	.	.	.	.	.
38-8	Do not offer continuous passive motion (CPM) (after total joint replacement surgery), pulsed electromagnetic field therapy, low-level laser therapy (LLLT), passive mobilizations, shock wave therapy, taping, thermotherapy, or ultrasound therapy to patients with hip or knee OA.	Str	1	1	.	.	.	.	.	.	.

QI v7_11: proportion using therapeutic ultrasound

Comment: 2 for & 5 against; joints 4 knee, 4 hip, 1 poly

id	verbatim_guideline_rec	LVL	Knee	Hip	Hand	Poly	Shldr	CV	GI	Frail	WPD
2-146	Level 5 strong recommendation against Therapeutic Ultrasound	Str	.	1	.	.	.	.	.	.	.
2-187	Level 4A conditional recommendation against Therapeutic Ultrasound	Cond	.	.	.	1	.	.	.	.	.
2-46	Level 4A conditional recommendation against Therapeutic Ultrasound	Cond	1	.	.	.	.	.	.	.	.
20-2	Physical passive interventions of manual therapy, ultrasound, acupuncture (high dose at least 3 times per week), and low-level laser therapy are recommended, based on current evidence, for knee OA.	NS	1	.	.	.	.	.	.	.	.
33-4	Clinicians may use ultrasound (1 MHz; 1 W/cm2 for 5 minutes each to the anterior, lateral, and posterior hip for a total of 10 treatments over a 2-week period) in addition to exercise and hot packs in the short-term management of pain and activity limitation in individuals with hip osteoarthritis	NS	.	1	.	.	.	.	.	.	.
36-52	We suggest not offering therapeutic ultrasound for people with knee and/or hip OA	Cond	1	1	.	.	.	.	.	.	.
38-8	Do not offer continuous passive motion (CPM) (after total joint replacement surgery), pulsed electromagnetic field therapy, low-level laser therapy (LLLT), passive mobilizations, shock wave therapy, taping, thermotherapy, or ultrasound therapy to patients with hip or knee OA.	Str	1	1	.	.	.	.	.	.	.

Domain: 08 Education

QI v8_1: proportion receiving education

Comment: 11 knee, 9 hip, 8 hand; 1 poly; 1 shoulder; 1 ankle; 1 wrist; comorb not specified

id	verbatim_guideline_rec	LVL	Knee	Hip	Hand	Poly	Shldr	CV	GI	Frail	WPD
18-2	Every patient should be provided with information regarding the disease and its course, self-management principles and therapeutic options.	NS	.	.	1	.	.	.	.	.	.
18-6	Every patient should be educated and trained in terms of ergonomic principles, activity breaks, and use of assistive devices	NS	.	.	1	.	.	.	.	.	.
2-1	CORE strong recommendation for arthritis education	Str	1	.	.	.	.	.	.	.	.
2-151	CORE strong recommendation for Arthritis Education	Str	.	.	.	1	.	.	.	.	.
2-80	CORE strong recommendation for Arthritis Education	Str	.	1	.	.	.	.	.	.	.
21-3	The patient should receive education	Str	1	1	1	.	.	.	.	.	.
24-7	Education and training in ergonomic principles, pacing of activity and use of assistive devices should be offered to every patient	NS	.	.	1	.	.	.	.	.	.
28-22	Information and education regarding treatment goals and the importance of lifestyle changes to reduce the degenerative damage of the knee joint should be provided.	Str	1	.	.	.	.	.	.	.	.
28-38	Information and education regarding the therapeutic objectives and the importance of changes in lifestyle, which include an exercise regimen, weight reduction, use of walking aids (walking stick and crutches) and shoe adjustments and other measures to prevent the progression of joint damage	Str	.	1	.	.	.	.	.	.	.
3-17	People with hip and/or knee OA should be taught a regular individualized (daily) exercise regimen and participate in self-management programs, strengthening, low-impact aerobic exercises, and neuromuscular education	NS	1	1	.	.	.	.	.	.	.
3-28	Information, education and an individually tailored program, including long-term and short-term goals, intervention or action plans to reduce the degenerative damage of the OA should be provided	NS	1	1	1	.	.	.	.	.	.
31-5	All patients with knee OA should be informed about the disease and its treatment.	NS	1	.	.	.	.	.	.	.	.
35-5	It is recommended to carry out health education for OA patients, mainly to educate them about the causes, prevention, progress and treatment of the disease, reduce the burden of patients' thoughts, and improve their self-management efficiency (1B)	Str	1	1	1	.	.	.	.	.	.
38-3	Consider limiting exercise therapy in the preoperative phase, teaching the patient exercises that he/she can independently perform, and monitoring how the exercises are performed if the risk of delayed postoperative recovery is not increased. Teach all patients to use a walking aid that will be needed in the postoperative phase.	Cond	1	1	.	.	.	.	.	.	.
4-1	A strong recommendation to the application of a core set comprising: information access/education, which is applicable throughout the management of knee OA	Str	1	.	.	.	.	.	.	.	.
40-1	Offer advice on access to appropriate information to all people with clinical osteoarthritis	Str	1	1	1	.	.	.	.	.	.
40-2	Offer accurate verbal and written information to all people with osteoarthritis to enhance understanding of the condition and its management, and to counter misconceptions, such as that it inevitably progresses and cannot be treated. Ensure that information sharing is an ongoing, integral part of the management plan rather than a single event at time of presentation	Str	1	1	1	.	1	.	.	.	.

Domain: 09 Behavioral/psychosocial

QI v9_1: proportion receiving psychological care

Comment: 2 for (depression); joints 2 knee, 2 hip, 1 hand, 1 wrist

id	verbatim_guideline_rec	LVL	Knee	Hip	Hand	Poly	Shldr	CV	GI	Frail	WPD
21-2	If indicated, the patient should receive psychological or social interventions	Str	1	1	1	.	.	.	.	.	.
41-7	Patients pending surgery who fall into depression or have catastrophic thoughts should be administered psychological/psychiatric assistance/care for the treatment of these problems (LE 1a; LR A; LA 88%).	Str	1	1	.	.	.	.	.	.	1

QI v9_2: proportion receiving vocational rehabilitation

Comment: 1 for; joints 1 knee, 1 hip

id	verbatim_guideline_rec	LVL	Knee	Hip	Hand	Poly	Shldr	CV	GI	Frail	WPD
3-8	People with hip or knee OA at risk of work disability should have access to vocational rehabilitation, including counselling about modifiable work-related factors	NS	1	1	.	.	.	.	.	.	.

QI v9_3: proportion using Cognitive Behavioral Therapy

Comment: 5 for (widespread pain) & 2 against (CV/GI/Frail); joints 3 knee, 4 hip, 1 hand, 2 poly

id	verbatim_guideline_rec	LVL	Knee	Hip	Hand	Poly	Shldr	CV	GI	Frail	WPD
1-44	Cognitive behavioral therapy (CBT) is conditionally recommended for patients with knee, hip, and/or hand OA.	Cond	1	1	1	.	.	.	.	.	.
2-167	Level 1B conditional recommendation for Cognitive Behavioral Therapy	Cond	.	.	.	1	.	.	.	.	1
2-168	Level 3/4B conditional recommendation against Cognitive Behavioral Therapy with or without exercise	Cond	.	.	.	1	.	1	1	1	.
2-19	Level 1B conditional recommendation for Cognitive Behavioral Therapy (with or without Exercise)	Cond	1	.	.	.	.	.	.	.	1
2-92	Level 2 conditional recommendation for Cognitive Behavioral Therapy	Cond	.	1	.	.	.	.	.	.	1
2-93	Level 3/4B conditional recommendation against Cognitive Behavioral Therapy alone	Cond	.	1	.	.	.	1	1	1	.
36-16	It may be appropriate to offer CBT for some people with knee and/or hip OA	Cond	1	1	.	.	.	.	.	.	.

Domain: 10 Procedures

QI v10_1: proportion referred to arthroscopic procedures

Comment: 1 for & 5 against; joints 6 knee

id	verbatim_guideline_rec	LVL	Knee	Hip	Hand	Poly	Shldr	CV	GI	Frail	WPD
16-1	Arthroscopic debridement and/or lavage of the knee joint has not been shown to have any beneficial effect on the natural history of OA, nor are these procedures indicated as a primary treatment in the management of OA of the knee.	NS	1	.	.	.	.	.	.	.	.
28-27	There is no benefit associated with the use of arthroscopy in the treatment of knee OA, even in the presence of a partial meniscal tear	Str	1	.	.	.	.	.	.	.	.
35-8	For knee OA patients with poor pain treatment response and mechanical symptoms, we recommend arthroscopy to reduce symptoms after assessing the risk of surgery (2C)	NS	1	.	.	.	.	.	.	.	.
36-15	We do not recommend offering arthroscopic, lavage and debridement, meniscectomy and cartilage repair for people with knee osteoarthritis (OA) unless the person also has mechanical symptoms of a clinically locked knee as per Australian Knee Society's 'Arthroscopy position statement'.	Str	1	.	.	.	.	.	.	.	.
37-1	We make a strong recommendation against the use of arthroscopy in nearly all patients with degenerative knee disease	Str	1	.	.	.	.	.	.	.	.
40-16	Do not refer for arthroscopic lavage and debridement as part of treatment for osteoarthritis, unless the person has knee osteoarthritis with a clear history of mechanical locking	Str	1	.	.	.	.	.	.	.	.

QI v10_2: proportion referred to surgery

Comment: 10 for; joints 6 knee, 4 hip, 4 hand

id	verbatim_guideline_rec	LVL	Knee	Hip	Hand	Poly	Shldr	CV	GI	Frail	WPD
18-3	Surgical interventions may be considered in patients with structural abnormalities when other treatment modalities are inadequate to alleviate the pain.	NS	.	.	1	.	.	.	.	.	.
24-1	Surgery should be considered for patients with structural abnormalities when other treatment modalities have not been sufficiently effective in relieving pain. Trapeziectomy should be considered in patients with thumb base OA and arthrodesis or arthroplasty in patients with interphalangeal OA	NS	.	.	1	.	.	.	.	.	.
28-10	Trapeziectomia, arthroplasty with ligament reconstruction and tendon interposition, or arthrodesis may be considered for severe OA of the base of the first finger (rhizarthrosis) if severe pain and/or disability and after conservative treatment have failed	Cond	.	.	1	.	.	.	.	.	.
28-2	Total hip arthroplasty is indicated when OA is accompanied by pain and walking difficulty and when the quality of life is impaired.	Str	.	1	.	.	.	.	.	.	.
3-6	Orthopedic surgery should be considered in patients with radiographic evidence of OA, who have marked disability, reduced quality of life and pain refractory to other treatments	NS	1	1	1	.	.	.	.	.	.
31-17	Total knee arthroplasty (TKA) should be considered in patients with advanced knee OA, who are resistant to pharmacological and non-pharmacological treatments and having pain, functional limitation and deterioration in daily living of life.	NS	1	.	.	.	.	.	.	.	.
31-2	Osteotomy, as a biomechanical correcting and preservative surgical method, can be advised to middle-aged and active patients with malalignment for patients with moderate-severe symptoms, functional capacity of either normal or minimally limited and/or radiologic grade of 2-3	NS	1	.	.	.	.	.	.	.	.
35-10	for patients with hip or knee OA who have poor response to conservative treatment and whose quality of life is significantly affected, we recommend to perform joint replacement after assessing the risk of surgery, which can relieve pain, increase the range of joint movement, and improve quality of life (1B)	Str	1	1	.	.	.	.	.	.	.
4-11	A strong recommendation to total knee replacement surgery for end-stage knee OA patients, which is a highly selective and cost-effective procedure although not devoid of adverse outcomes; the role of other surgical procedures, especially unicompartmental knee replacement, should be further investigated.	Str	1	.	.	.	.	.	.	.	.
40-29	Consider referral for joint surgery for people with osteoarthritis who experience joint symptoms (pain, stiffness and reduced function) that have a substantial impact on their quality of life and are refractory to non-surgical treatment	NS	1	1	.	.	.	.	.	.	.

Domain: 11 Other

QI v11_1: proportion receiving an annual evaluation

Comment: 1 for; joints 1 knee, 1 hip, 1 hand, 1 shoulder, 1 ankle

id	verbatim_guideline_rec	LVL	Knee	Hip	Hand	Poly	Shldr	CV	GI	Frail	WPD
40-5	Consider an annual review for any person with one or more of the following: ï¼troublesome joint pain ï¼more than one joint with symptoms ï¼more than one comorbidity ï¼taking regular medication for their osteoarthritis.	Cond	1	1	1	.	1	.	.	.	.

QI v11_2: proportion receiving routine imaging to monitor OA

Comment: 3 against; joints 3 knee, 3 hip, 3 hand

id	verbatim_guideline_rec	LVL	Knee	Hip	Hand	Poly	Shldr	CV	GI	Frail	WPD
29-1	Routine imaging in OA follow-up is not recommended. However, imaging is recommended if there is unexpected rapid progression of symptoms or change in clinical characteristics to determine if this relates to OA severity or an additional diagnosis.	NS	1	1	1	.	.	.	.	.	.
29-2	According to current evidence, imaging features do not predict non-surgical treatment response and imaging cannot be recommended for this purpose.	NS	1	1	1	.	.	.	.	.	.
3-18	Imaging features do not predict non-surgical treatment response.	NS	1	1	1	.	.	.	.	.	.

QI v11_3: proportion using ultrasound to monitor OA

Comment: 1 against; joints 1 knee, 1 hip, 1 hand

id	verbatim_guideline_rec	LVL	Knee	Hip	Hand	Poly	Shldr	CV	GI	Frail	WPD
26-3	Ultrasound should not be used as a routine imaging in the follow-up and prognosis of osteoarthritis.	NS	1	1	1	.	.	.	.	.	.

Domain: 12 Exercise

QI v12_1: proportion receiving exercise + education

Comment: 10 for; joints 7 knee, 2 hip, 1 hand

id	verbatim_guideline_rec	LVL	Knee	Hip	Hand	Poly	Shldr	CV	GI	Frail	WPD
10-1	The Ottawa Panel strongly recommends 12 weeks of supervised group strength training, functional, and flexibility exercises with patient education programme for hip osteoarthritis management and improvement of physical function	Str	.	1	.	.	.	.	.	.	.
12-18	A 12-week strengthening exercise program with home exercises and usual care, including patient education and medication if necessary (exercises for muscle functions, mobility and coordination and instructions) (one to three days per week) for the management of elderly individuals with knee osteoarthritis for pain relief (VAS) at 12 weeks end of treatment is suggested.	NS	1	.	.	.	.	.	.	.	.
12-20	A four-week group education program followed by eight weeks of the unsupervised homebased exercise program (active range-of-motion exercises, muscle strengthening and muscle stretching) (45 minutes one day per week) for the management of knee osteoarthritis for pain relief (WOMAC subscale) and improved physical function (WOMAC subscale) after those eight weeks of home exercises is recommended.	NS	1	.	.	.	.	.	.	.	.
12-22	A eight-week strengthening exercise program (stretching, strength, mobility, functional, balance, relaxation) with patient education (60 minute sessions completed twice weekly) for the management of adults with knee osteoarthritis for pain relief (Lequesne Index (LI) subscale) and suggests its use for improved physical function (LI subscale) at eight weeks end of treatment is recommended.	NS	1	.	.	.	.	.	.	.	.
12-3	A six-week progressive exercise program (warm-up, strengthening, balance and motor control exercises), education and usual care (45 minutes, two times per week), either in a group or individually, for the management of knee osteoarthritis for improved physical function (WOMAC subscale) at the end of treatment of six weeks is strongly recommended.	NS	1	.	.	.	.	.	.	.	.
12-8	A six-week osteoarthritis education and supervised strengthening exercise program with home exercises (resistance exercises for the knee and hip independently of the site of major pain) (one two-hour session per week) for the management of adults with knee osteoarthritis for pain relief (Visual Analogue Scale (VAS)) at the six-month follow-up is suggested.	NS	1	.	.	.	.	.	.	.	.
13-1	A three-month aerobic, strengthening exercise program and osteoarthritis health education (brisk walking, isometric and isotonic muscle strengthening with therapeutic elastic bands, stretching) (one hour sessions, three times per week) for the management of knee osteoarthritis for pain relief during weight-bearing activities (Abnormal Involuntary Movement Scale 2 (AIMS2) subscale) and improved physical function (AIMS2 subscale) at the end of treatment of three months is strongly recommended.	NS	1	.	.	.	.	.	.	.	.
28-46	Education with regard to joint protection together with an exercise regimen including muscle strengthening and range of motion exercises.	Str	.	.	1	.	.	.	.	.	.
33-9	Clinicians should provide patient education combined with exercise and/or manual therapy.	NS	.	1	.	.	.	.	.	.	.
6-2	To provide pain relief, manage the symptoms and delay the progression of the disease, patients should understand their conditions from their doctor and routinely perform moderate exercises.	NS	1	.	.	.	.	.	.	.	.

QI v12_2: proportion receiving mobilization and manipulation

Comment: 8 for & 5 against; joints 8 knee, 8 hip, 1 poly

id	verbatim_guideline_rec	LVL	Knee	Hip	Hand	Poly	Shldr	CV	GI	Frail	WPD
1-28	Manual therapy with exercise is conditionally recommended against over exercise alone in patients with knee and/or hip OA.	Cond	1	1	.	.	.	.	.	.	.
2-113	Level 4A conditional recommendation against Mobilization & Manipulation	Cond	.	1	.	.	.	.	.	.	.
2-186	Level 4A conditional recommendation against Mobilization & Manipulation	Cond	.	.	.	1	.	.	.	.	.
2-42	Level 4A conditional recommendation against Mobilization & Manipulation	Cond	1	.	.	.	.	.	.	.	.
20-2	Physical passive interventions of manual therapy, ultrasound, acupuncture (high dose at least 3 times per week), and low-level laser therapy are recommended, based on current evidence, for knee OA.	NS	1	.	.	.	.	.	.	.	.
20-3	Physical passive interventions of manual therapy, based on current evidence, for hip OA.	NS	.	1	.	.	.	.	.	.	.
28-20	Mechanotherapy, including flexibility programs and mobilization and stretching exercises, can reduce pain and improve the range of motion of the knee	Cond	1	.	.	.	.	.	.	.	.
33-5	Clinicians should use manual therapy for patients with mild to moderate hip osteoarthritis and impairment of joint mobility, flexibility, and/or pain. Manual therapy may include thrust, nonthrust, and soft tissue mobilization	NS	.	1	.	.	.	.	.	.	.
35-1	Physical therapy such as manipulation therapy, massage, and acupuncture is recommended for OA patients to relieve pain and improve physical function (2B)	NS	1	1	.	.	.	.	.	.	.
36-20	It may be appropriate to offer a short course of manual therapy (stretching, soft tissue and/or joint mobilization and/or manipulation) for some people with knee and/or hip OA	Cond	1	1	.	.	.	.	.	.	.
38-8	Do not offer continuous passive motion (CPM) (after total joint replacement surgery), pulsed electromagnetic field therapy, low-level laser therapy (LLLT), passive mobilizations, shock wave therapy, taping, thermotherapy, or ultrasound therapy to patients with hip or knee OA.	Str	1	1	.	.	.	.	.	.	.
4-8	In Step 1 background treatment, if symptomatic after referral to physical therapist for knee braces and insoles, the following can be added at any time: Walking aids, thermal agents, mechanotherapy, bandage tape, hydrotherapy and aquatic exercises, and tai chi	NS	1	.	.	.	.	.	.	.	.
40-8	Manipulation and stretching should be considered as an adjunct to core treatments, particularly for osteoarthritis of the hip.	Cond	.	1	.	.	.	.	.	.	.

QI v12_3: proportion receiving weight management

Comment: 11 for & 2 against (frail); joints 10 knee, 9 hip, 3 hand, 2 poly, 1 shoulder, 1 ankle, 1 wrist

id	verbatim_guideline_rec	LVL	Knee	Hip	Hand	Poly	Shldr	CV	GI	Frail	WPD
1-7	Weight loss is strongly recommended for patients with knee and/or hip OA who are overweight or obese.	Str	1	1	.	.	.	.	.	.	.
2-164	Level 1B conditional recommendation for Dietary Weight Management (with or without Exercise)	Cond	.	.	.	1	.	1	1	.	1
2-165	Level 3/4B conditional recommendation against Dietary Weight Management with or without exercise	Cond	.	.	.	1	.	.	.	1	.
2-99	Level 3/4B conditional recommendation against Dietary Weight Management with or without exercise	Cond	.	1	.	.	.	.	.	.	.
21-4	If indicated, the patient should receive weight management	Str	1	1	1	.	.	.	.	.	.
3-13	Patients with hip and knee OA, who are overweight, should be encouraged to lose weight and maintain their weight at a lower level	NS	1	1	.	.	.	.	.	.	.
31-3	People with symptomatic knee osteoarthritis and body mass index of equal to and greater than 25 should be advised to lose weight	NS	1	.	.	.	.	.	.	.	.
35-16	It is recommended that OA patients should control their weight, and those who are overweight or obese should lose weight (1A)	Str	1	1	.	.	.	.	.	.	.
36-5	We strongly recommend weight management for people with knee and/or hip OA. For those who are overweight (BMI â‰¥25 kg/m2) or obese (BMI â‰¥30 kg/m2), a minimum weight loss target of 5-7.5% of body weight is recommended.	Str	1	1	.	.	.	.	.	.	.
4-9	A strong recommendation to the application of a core set comprising: weight loss, which is applicable throughout the management of knee OA	Str	1	.	.	.	.	.	.	.	.
40-30	Offer advice on interventions to achieve weight loss to all people with clinical osteoarthritis if the person is overweight or obese	Str	1	1	1	.	.	.	.	.	.
40-9	Offer interventions to achieve weight loss as a core treatment for people who are obese or overweight	Str	1	1	1	.	1	.	.	.	.
41-1	The recommendation is that patients pending surgery should control their weight and maintain the highest possible level of physical activity, symptoms permitting. Obese patients in particular should try to lose weight (LE 2a; LR B; LA 100%).	Cond	1	1	.	.	.	.	.	.	.

QI v12_4: proportion receiving weight management + exercise

Comment: 3 for; joints 2 knee, 2 hip

id	verbatim_guideline_rec	LVL	Knee	Hip	Hand	Poly	Shldr	CV	GI	Frail	WPD
12-4	A 24-month quadriceps strengthening exercise program (with graded therapeutic elastic bands exercises; flexibility exercises; functional strengthening exercises; stretching exercises (twice daily) plus a dietary intervention) for the management of overweight adults with knee osteoarthritis for pain relief (WOMAC subscale) at 24 months end of treatment is strongly recommended.	NS	1	.	.	.	.	.	.	.	.
33-2	In addition to providing exercise intervention, clinicians should collaborate with physicians, nutritionists, or dietitians to support weight reduction in individuals with hip osteoarthritis who are overweight or obese.	NS	.	1	.	.	.	.	.	.	.
36-21	It may be appropriate to offer a combination of weight management plus exercise for some people with knee and/or hip OA. For those who are overweight (BMI â‰¥25 kg/m2) or obese (BMI â‰¥30 kg/m2), a minimum weight loss target of 5-7.5% of body weight is recommended.	Cond	1	1	.	.	.	.	.	.	.

QI v12_5: proportion using Cognitive Behavioral Therapy + exercise

Comment: 1 for (CV/GI/Frail) & 2 against (Widspread pain); joints 1 knee, 1 hip, 1 poly

id	verbatim_guideline_rec	LVL	Knee	Hip	Hand	Poly	Shldr	CV	GI	Frail	WPD
2-169	Level 3 conditional recommendation against Cognitive Behavioral Therapy with Exercise	Cond	.	.	.	1	.	.	.	.	1
2-18	Level 2 conditional recommendation for Cognitive Behavioral Therapy with Exercise	Cond	1	.	.	.	.	1	1	1	.
2-94	Level 4A conditional recommendation against Cognitive Behavioral Therapy with Exercise	Cond	.	1	.	.	.	.	.	.	.

QI v12_6: proportion using aquatic exercise

Comment: 6 for (knee) & 2 against; joints 6 knee, 3 hip, 1 hand, 1 poly

id	verbatim_guideline_rec	LVL	Knee	Hip	Hand	Poly	Shldr	CV	GI	Frail	WPD
2-109	Level 4A conditional recommendation against Aquatic Exercise	Cond	.	1	.	.	.	.	.	.	.
2-15	Level 1B conditional recommendation for Aquatic Exercise	Cond	1	.	.	.	.	.	.	.	.
2-171	Level 3/4B/4A conditional recommendation against Aquatic Exercise	Cond	.	.	.	1	.	.	.	.	.
3-11	Patients with knee OA should participate in aerobic and/or resistance land-based and/or aquatic exercise	NS	1	.	.	.	.	.	.	.	.
3-16	Balneotherapy and exercises in water are effective for relieving symptoms in hip and knee (and hand) OA	NS	1	1	1	.	.	.	.	.	.
36-18	It may be appropriate to offer aquatic exercise/hydrotherapy for some people with knee and/or hip OA	Cond	1	1	.	.	.	.	.	.	.
4-8	In Step 1 background treatment, if symptomatic after referral to physical therapist for knee braces and insoles, the following can be added at any time: Walking aids, thermal agents, mechanotherapy, bandage tape, hydrotherapy and aquatic exercises, and tai chi	NS	1	.	.	.	.	.	.	.	.
6-9	The following exercises under supervision are recommended: straight leg raises, jogging, cycling, swimming, Tai Chi and Ba Duan Jin exercise	NS	1	.	.	.	.	.	.	.	.

QI v12_7: proportion using exercise

Comment: 28 for; joints 19 knee, 18 hip, 7 hand, 1 poly, 1 shoulder, 1 ankle, 1 wrist

id	verbatim_guideline_rec	LVL	Knee	Hip	Hand	Poly	Shldr	CV	GI	Frail	WPD
1-21	Exercise is strongly recommended for patients with knee, hip, and/or hand OA.	Str	1	1	1	.	.	.	.	.	.
10-2	The Ottawa Panel strongly recommends eight weeks of strength training and flexibility exercises for hip osteoarthritis management of physical function	Str	.	1	.	.	.	.	.	.	.
10-3	Therapeutic exercise, especially strength training, can greatly improve pain, disability, physical function, stiffness, and range of motion for hip osteoarthritis patients.	NS	.	1	.	.	.	.	.	.	.
10-6	Flexibility exercises have shown great improvements for pain, range of motion, physical function, and stiffness.	NS	.	1	.	.	.	.	.	.	.
12-19	A eight-week concentric-eccentric isokinetic or isometric exercise programs + paracetamol (cycling warm-up before the exercises; application of isokinetic dynamometer for exercises; cool-down after the exercises) (three days weekly) for the management of knee osteoarthritis for pain relief during rest (Visual Analogue Scale (VAS)), pain relief during motion (VAS), improved physical function (Western Ontario and McMaster Universities Arthritis Index (WOMAC) subscale) and improved quality of life (Short Form 36 (SF-36)) subscale) at eight-weeks end of treatment and at 12-weeks follow-up is recommended.	NS	1	.	.	.	.	.	.	.	.
12-21	An eight-week high or low-resistance strengthening exercise program (10% and 60% 1-RM leg training) (both high and low-resistance groups completed it three times weekly) for the management of knee osteoarthritis for pain relief (WOMAC subscale) and improved physical function (Western Ontario and McMaster Universities Arthritis Index (WOMAC) subscale) at eight weeks end of treatment is strongly recommended.	NS	1	.	.	.	.	.	.	.	.
14-1	A wide variety of programmes involving therapeutic exercise seem to be beneficial for hand osteoarthritis management, but isolation of the effect of exercise components requires further research.	NS	.	.	1	.	.	.	.	.	.
18-5	Exercise should be considered for all patients to enhance function and muscle strength in addition to reducing pain	NS	.	.	1	.	.	.	.	.	.
2-152	CORE strong recommendation for Structured Exercise Programs (Type 1- strengthening and/or cardio and/or balance training/neuromuscular)	Str	.	.	.	1	.	.	.	.	.
2-81	CORE strong recommendation for Structured Exercise Programs (Type 1- strengthening and/or cardio and/or balance training/neuromuscular)	Str	.	1	.	.	.	.	.	.	.
20-1	Physical active interventions of exercise are recommended, based on current evidence, for knee and hip OA.	NS	1	1	.	.	.	.	.	.	.
21-7	If indicated, the patient should receive physical activity and exercise.	Str	1	1	1	.	.	.	.	.	.
23-3	Adapted physical activity (APA) and structured therapeutic exercise (STE) can limit knee and hip OA symptoms and progression	NS	1	1	.	.	.	.	.	.	.
24-9	Exercises to improve function and muscle strength, as well as to reduce pain, should be considered for every patient	NS	.	.	1	.	.	.	.	.	.
27-1	Modes of delivery. Healthcare providers should consider different modes of delivery of physical activity (PA) (eg, supervised/not-supervised, individual/group, face-to-face/online, booster strategies) in line with people's preferences	Cond	1	1	.	.	.	.	.	.	.
27-3	PA as integral part of standard care. Promoting physical activity (PA) consistent with general PA recommendations should be an integral part of standard care throughout the course of disease	Str	1	1	.	.	.	.	.	.	.
27-5	Responsibility for physical activity (PA) promotion. All healthcare providers involved in the management of people with RA/SpA/HOA/KOA should take responsibility for promoting PA and should cooperate, including making	Cond	1	1	.	.	.	.	.	.	.

	necessary referrals, to ensure that people with RA/SpA/HOA/KOA receive appropriate PA-interventions.										
27-7	Individual adaptations to PA following individualised assessment. Where individual adaptations to general PA recommendations are needed, these should be based on a comprehensive assessment of physical, social and psychological factors including fatigue, pain, depression and disease activity. General and disease-specific contraindications for PA should be identified and taken into account in the promotion of PA.	Cond	1	1	.	.	.	.	.	.	.
27-8	General and disease-specific barriers and facilitators related to performing physical activity (PA), including knowledge, social support, symptom control and self-regulation should be identified and addressed	Cond	1	1	.	.	.	.	.	.	.
28-28	Exercises for concentric contraction of the flexor and extensor muscles of the knee are indicated as these have been shown to reduce pain both at rest and during activity	Str	1	.	.	.	.	.	.	.	.
3-17	People with hip and/or knee OA should be taught a regular individualized (daily) exercise regimen and participate in self-management programs, strengthening, low-impact aerobic exercises, and neuromuscular education	NS	1	1	.	.	.	.	.	.	.
31-11	All patients should be evaluated individually and appropriate exercise programme should be planned accordingly	NS	1	.	.	.	.	.	.	.	.
33-1	Clinicians should use individualized flexibility, strengthening, and endurance exercises to address impairments in hip range of motion, specific muscle weaknesses, and limited thigh (hip) muscle flexibility.	NS	.	1	.	.	.	.	.	.	.
35-6	It is recommended for OA patients to choose different activities according to the location of the disease such as grasping and holding activities of hand joints, flexion and extension activities of knee joints under the condition of non-load, and gentle activities in different directions of cervical and lumbar joints (1B)	Str	1	.	.	.	.	.	.	.	.
38-1	Offer exercise therapy to all patients with OA of the knee or hip in the conservative treatment phase, and make use of the frequency, intensity, time, and type (FITT) principles.	Str	1	1	.	.	.	.	.	.	.
4-4	A strong recommendation to the application of a core set comprising: an exercise program, which is applicable throughout the management of knee OA	Str	1	.	.	.	.	.	.	.	.
40-31	Offer advice on activity and exercise to all people with clinical osteoarthritis	Str	1	1	1	.	.	.	.	.	.
40-4	Advise people with osteoarthritis to exercise as a core treatment, irrespective of age, comorbidity, pain severity or disability. Exercise should include: ï¼local muscle strengthening and ï¼general aerobic fitness.	Str	1	1	1	.	1	.	.	.	.

QI v12_8: proportion using land-based exercise

Comment: 19 for; joints 17 knee, 3 hip

id	verbatim_guideline_rec	LVL	Knee	Hip	Hand	Poly	Shldr	CV	GI	Frail	WPD
1-18	Balance exercises are conditionally recommended for patients with knee and/or hip OA.	Cond	1	1	.	.	.	.	.	.	.
12-15	A eight-week concentric-eccentric quadriceps strengthening exercise program (concentric quadriceps action followed by an eccentric quadricep action) (50 minute classes three times a week) for the management of knee osteoarthritis for pain relief (WOMAC subscale) and improved physical function (WOMAC subscale) at eight weeks end of treatment is recommended.	NS	1	.	.	.	.	.	.	.	.
12-16	12-week progressive resistance exercise program of knees and hip muscles (with machines and free weights) (two days per week) for the management of women with knee osteoarthritis for pain relief (Visual Analogue Scale (VAS)), pain relief (Western Ontario and McMaster Universities Arthritis Index (WOMAC) subscale), improved physical function (WOMAC subscale), improved physical function (Short Form 36 (SF-36) subscale) and improved quality of life (SF-36 subscale) at 90 days end of treatment is recommended.	NS	1	.	.	.	.	.	.	.	.
12-17	A 12-week progressive hip muscle strengthening home-based exercise program (isotonic resisted with ankle weights or therapeutic elastic bands) (five days per week at home plus seven physiotherapy consultations of 15ï¿½30 minutes during two months) for medial knee osteoarthritis management for pain relief (WOMAC subscale) and improved physical function (WOMAC subscale) at the one-week follow-up is recommended.	NS	1	.	.	.	.	.	.	.	.
12-2	A four-month home-based progressive strengthening exercise program (isotonic and resistance exercises of hips and knees with ankle weights and functional strengthening) (three days per week) for knee osteoarthritis management for pain relief (Western Ontario and McMaster Universities Arthritis Index (WOMAC) subscale) and improved physical function (WOMAC subscale) at four months end of treatment is recommended.	NS	1	.	.	.	.	.	.	.	.
12-23	A three-month strengthening (all Lower Extremity (LE) muscles with ankle weights, therapeutic elastic bands, non-weight-bearing and weight-bearing) and balance (gait training) exercise program (twice weekly) for knee osteoarthritis management for improved physical function (usual walking speed) at three months end of treatment is recommended	NS	1	.	.	.	.	.	.	.	.
12-7	A six-month lower extremity strengthening exercise program (isometric, isotonic and dynamic exercises) (one set of exercises performed daily) for the management of knee osteoarthritis for improved quality of life (Hospital Anxiety and Depression Scale (HADS) subscale) at six months end of treatment is strongly recommended.	NS	1	.	.	.	.	.	.	.	.
13-3	A 12-week cycling exercise program (warm-up, aerobic loading, cool-down) (20-60-minute classes, two-six days per week) for the management of knee osteoarthritis for pain relief (WOMAC subscale) and improved physical function (WOMAC subscale) is recommended	NS	1	.	.	.	.	.	.	.	.
13-4	A short-term aerobic exercise program with or without strengthening exercises is promising for reducing pain, as well as improving physical function and quality of life for individuals with knee osteoarthritis.	NS	1	.	.	.	.	.	.	.	.
2-2	CORE strong recommendation for Structured Land-Based Exercise Programs (Type 1- strengthening and/or cardio and/or balance training/neuromuscular exercise) with or without Dietary Weight Management	Str	1	.	.	.	.	.	.	.	.
28-39	A program of flexibility, stretching, and strengthening exercises for symptomatic knee OA is recommended as this reduces pain during walking and climbing stairs and improves the strength of the quadriceps femoris.	Str	1	.	.	.	.	.	.	.	.
28-58	Aerobic exercise can be implemented gradually and progressively according to each patient's level of fitness at a frequency of three or more times per week, with a minimum duration of 20 to 30 minutes per session	Str	1	.	.	.	.	.	.	.	.

28-6	A daily walk is recommended as this improves muscle strength, aerobic capacity, and endurance; facilitates a good night's sleep; and reduces knee pain	Str	1	.	.	.	.	.	.	.	.
28-64	Aerobic exercise performed on a regular basis and muscle stretching and strengthening and joint mobility exercises are recommended	Str	.	1	.	.	.	.	.	.	.
3-11	Patients with knee OA should participate in aerobic and/or resistance land-based and/or aquatic exercise	NS	1	.	.	.	.	.	.	.	.
36-1	We strongly recommend offering land-based exercise for all people with knee OA to improve pain and function, regardless of their age, structural disease severity, functional status or pain levels. We strongly recommend walking, muscle-strengthening exercise, and specifically, Tai Chi.	Str	1	.	.	.	.	.	.	.	.
36-17	It may be appropriate to offer stationary cycling and/or Hatha yoga for some people with knee OA	Cond	1	.	.	.	.	.	.	.	.
36-3	We strongly recommend offering land-based exercise for all people with hip OA to improve pain and function, regardless of their age, structural disease severity, functional status or pain levels.	Str	.	1	.	.	.	.	.	.	.
6-9	The following exercises under supervision are recommended: straight leg raises, jogging, cycling, swimming, Tai Chi and Ba Duan Jin exercise	NS	1	.	.	.	.	.	.	.	.

QI v12_9: proportion using mind-body exercise (Yoga or Tai Chi)

Comment: 13 for; joints 11 knee, 2 hip, 1 poly

id	verbatim_guideline_rec	LVL	Knee	Hip	Hand	Poly	Shldr	CV	GI	Frail	WPD
1-45	Yoga is conditionally recommended for patients with knee OA.	Cond	1	.	.	.	.	.	.	.	.
1-5	Tai chi is strongly recommended for patients with knee and/or hip OA.	Str	1	1	.	.	.	.	.	.	.
11-1	The 12-week Sun style Tai Chi exercise program (60 minute classes once per week) for management of knee osteoarthritis	NS	1	.	.	.	.	.	.	.	.
11-2	The 20-week Sun style Tai Chi exercise program (20 to 40-minute classes three times per week) for the management of knee osteoarthritis for pain relief and improved physical function at the end of treatment (20 weeks) is strongly recommended.	Str	1	.	.	.	.	.	.	.	.
11-3	The eight-week Tai Chi Qigong program (60 minute classes twice per week) for the management of knee osteoarthritis for improved quality of life; suggested for pain relief	NS	1	.	.	.	.	.	.	.	.
11-4	The eight-week Hatha Yoga program (60 minute classes once per week, plus 30 minute home program four times per week) for older women; Suggested for improved physical function	NS	1	.	.	.	.	.	.	.	.
2-163	Level 1B conditional recommendation for Mind-body Exercise	Cond	.	.	.	1	.	.	.	.	.
2-3	CORE strong recommendation for Structured Land-Based Exercise Programs (Type 2- Mind-body Exercise including Tai Chi or Yoga) with or without Dietary Weight Management	Str	1	.	.	.	.	.	.	.	.
2-89	Level 1B conditional recommendation for Mind-body Exercise	Cond	.	1	.	.	.	.	.	.	.
36-1	We strongly recommend offering land-based exercise for all people with knee OA to improve pain and function, regardless of their age, structural disease severity, functional status or pain levels. We strongly recommend walking, muscle-strengthening exercise, and specifically, Tai Chi.	Str	1	.	.	.	.	.	.	.	.
36-17	It may be appropriate to offer stationary cycling and/or Hatha yoga for some people with knee OA	Cond	1	.	.	.	.	.	.	.	.
4-8	In Step 1 background treatment, if symptomatic after referral to physical therapist for knee braces and insoles, the following can be added at any time: Walking aids, thermal agents, mechanotherapy, bandage tape, hydrotherapy and aquatic exercises, and tai chi	NS	1	.	.	.	.	.	.	.	.
6-9	The following exercises under supervision are recommended: straight leg raises, jogging, cycling, swimming, Tai Chi and Ba Duan Jin exercise	NS	1	.	.	.	.	.	.	.	.

QI v12_10: proportion using minescectomy

Comment: 1 for ; joints 1 knee

id	verbatim_guideline_rec	LVL	Knee	Hip	Hand	Poly	Shldr	CV	GI	Frail	WPD
28-56	In patients with a partial rupture of the meniscus, a partial meniscectomy performed arthroscopically may be beneficial, followed by a physical therapy program	Cond	1	.	.	.	.	.	.	.	.

QI v12_11: proportion using physical therapy

Comment: 18 for; joints 14 knee, 6 hip, 1 hand, 1 shoulder

id	verbatim_guideline_rec	LVL	Knee	Hip	Hand	Poly	Shldr	CV	GI	Frail	WPD
1-31	Overall, exercise programs are more effective if supervised, often by physical therapists and sometimes in a class setting, rather than when performed by the individual at home.	NS	1	1	1	.	.	.	.	.	.
10-4	The Ottawa Panel strongly recommends 12 weeks of supervised group strength training and stretching exercises with general practitioner care for hip osteoarthritis management for pain and suggests its use for physical function	Str	.	1	.	.	.	.	.	.	.
10-5	The Ottawa Panel strongly recommends eight weeks of supervised group strength training in conjunction with unsupervised home exercises for hip osteoarthritis management for pain	Str	.	1	.	.	.	.	.	.	.
12-12	A three-month physiotherapy exercise program (strengthening/endurance, balance and stretching exercises) (50 minutes classes twice a week) for the management of knee osteoarthritis for pain relief at rest (Visual Analogue Scale (VAS)) at three-months follow-up is recommended.	NS	1	.	.	.	.	.	.	.	.
12-13	An eight-week supervised isokinetic, isotonic or isometric muscle strengthening exercise programs (warm-up stationary bike; Range of Motion (ROM) assessment in each arthritic joint; stretching; hot packs) (three days per week) for the management of bilateral knee osteoarthritis for pain relief (Visual Analogue Scale (VAS)) and for improved physical function (Lequesne Index (LI) subscale) at eight weeks end of treatment and at one-year follow-up is strongly recommended.	NS	1	.	.	.	.	.	.	.	.
12-25	A 12-week group-based supervised progressive strengthening and coordination exercise program (circuit training) (one hour sessions three days per week) for the management of knee osteoarthritis for improved quality of life (Knee injury and Osteoarthritis Outcome Score (KOOS) quality of life subscale) at 12 weeks end of treatment is suggested.	NS	1	.	.	.	.	.	.	.	.
12-26	A 12-week progressive supervised squat exercise program (bicycle warm-up; squat exercises with/without whole body vibration) (three days per week on alternate days) for the management of knee osteoarthritis for pain relief (Western Ontario and McMaster Universities Arthritis Index (WOMAC) subscale) and improved physical function (WOMAC subscale) at 12 weeks end of treatment is suggested.	NS	1	.	.	.	.	.	.	.	.
12-5	A two-week mechanical diagnosis and therapy exercise program (end-range exercises; advice on exercises for aerobic as well strengthening of the quadriceps; biking; walking) (10 repetitions every 2-3 hours) for the management of knee osteoarthritis for pain relief (P4 subscale), pain relief (Knee injury and Osteoarthritis Outcome Score (KOOS) pain subscale) and improved physical function (KOOS in daily living subscale) at two weeks end of treatment is strongly recommended.	NS	1	.	.	.	.	.	.	.	.
12-6	12-week home-based physiotherapist prescribed supervised quadriceps strengthening exercise program (using ankle weights, therapeutic elastic bands) (five days per week) for the management of knee osteoarthritis for pain relief (Western Ontario and McMaster Universities Arthritis Index (WOMAC) subscale) at 12 weeks end of treatment is strongly recommended.	NS	1	.	.	.	.	.	.	.	.
12-9	A three-month physiotherapy exercise interventions (mobility training, venous therapy, lower extremity and trunk muscle strengthening, flexibility, coordination and balance exercises) (twice weekly for three months) for the management of knee osteoarthritis for pain relief at night (Visual Analogue Scale (VAS)) at nine-months follow-up is recommended.	NS	1	.	.	.	.	.	.	.	.
13-2	A 10-week community physiotherapy exercise interventions (an individualised aerobic and strengthening exercise	NS	1	.	.	.	.	.	.	.	.

	program and advice leaflet about activity and pacing) (20 minutes, three-six times over 10 weeks) for the management of knee osteoarthritis for pain relief (WOMAC subscale) and improved physical function (Western Ontario and McMaster Universities Osteoarthritis (WOMAC) subscale) at the three months follow-up is strongly recommended.										
13-5	An eight-week individual or group supervised aerobic and strengthening exercise programs (running, eccentric and concentric exercises, stairs, stepper machine, home exercise program) (at the therapist discretion or one hour two times per week) for the management of knee osteoarthritis for pain relief (WOMAC subscale) at the end of treatment of eight weeks is strongly recommended.	NS	1	.	.	.	.	.	.	.	.
13-6	A four-week leg functional aerobic and strengthening exercise program (supervised exercise: riding a stationary bike, active range of motion for the knee, muscle strengthening exercises for the hip and knee, muscle stretching and manual physical therapy) (two 30-minute sessions per week) for the management of knee osteoarthritis for improved physical function (WOMAC subscale) at end of treatment of four weeks and at the four weeks follow-up is recommended.	NS	1	1	.	.	.	.	.	.	.
22-3	We suggest offering physical therapy as part of a comprehensive management plan for patients with osteoarthritis of the hip or knee.	Cond	1	1	.	.	.	.	.	.	.
34-2	In the absence of reliable evidence, it is the opinion of the work group that physical therapy may benefit select patients with glenohumeral joint osteoarthritis.	Cond	.	.	.	.	1	.	.	.	.
4-14	It was recommended that, in Step 1 of background treatment and after adhering to the basic principle and core set, patients should be referred to a physical therapist or another specialist for assessment of whether correction for varus/valgus malalignment is needed	NS	1	.	.	.	.	.	.	.	.
4-8	In Step 1 background treatment, if symptomatic after referral to physical therapist for knee braces and insoles, the following can be added at any time: Walking aids, thermal agents, mechanotherapy, bandage tape, hydrotherapy and aquatic exercises, and tai chi	NS	1	.	.	.	.	.	.	.	.
5-15	Strong evidence supports the use of physical therapy as a treatment to improve function and reduce pain for patients with osteoarthritis of the hip and mild to moderate symptoms.	Str	.	1	.	.	.	.	.	.	.

References

Ref ID	authors	title	journal	pub_year	pubmed_id	doi
1	Kolasinski SL, Neogi T, Hochberg MC, Oatis C, Guyatt G, Block J, Callahan L, Copenhaver C, Dodge C, Felson D, Gellar K, Harvey WF, Hawker G, Herzig E, Kwoh CK, Nelson AE, Samuels J, Scanzello C, White D, Wise B, Altman RD, DiRenzo D, Fontanarosa J, Giradi G, Ishimori M, Misra D, Shah AA, Shmagel AK, Thoma LM, Turgunbaev M, Turner AS, Reston J.	2019 American College of Rheumatology/Arthritis Foundation Guideline for the Management of Osteoarthritis of the Hand, Hip, and Knee	Arthritis Rheumatol	2020	31908163	10.1002/art.41142
2	Bannuru RR, Osani MC, Vaysbrot EE, Arden NK, Bennell K, Bierma-Zeinstra SMA, Kraus VB, Lohmander LS, Abbott JH, Bhandari M, Blanco FJ, Espinosa R, Haugen IK, Lin J, Mandl LA, Moilanen E, Nakamura N, Snyder-Mackler L, Trojian T, Underwood M, McAlindon TE.	OARSI guidelines for the non-surgical management of knee, hip, and polyarticular osteoarthritis	Osteoarthritis Cartilage	2019	31278997	10.1016/j.joca.2019.06.011
3	Ariani A, Manara M, Fioravanti A, Iannone F, Salaffi F, Ughi N, Prevete I, Bortoluzzi A, Parisi S, Scirï¿½ CA.	The Italian Society for Rheumatology clinical practice guidelines for the diagnosis and management of knee, hip and hand osteoarthritis	Reumatismo	2019	31948191	10.4081/reumatismo.2019.1188
4	Bruyere O, Honvo G, Veronese N, Arden NK, Branco J, Curtis EM, Al-Daghri NM, Herrero-Beaumont G, Martel-Pelletier J, Pelletier JP, Rannou F, Rizzoli R, Roth R, Uebelhart D, Cooper C, Reginster JY.	An updated algorithm recommendation for the management of knee osteoarthritis from the European Society for Clinical and Economic Aspects of Osteoporosis, Osteoarthritis and Musculoskeletal Diseases (ESCEO)	Semin Arthritis Rheum	2019	31126594	10.1016/j.semarthrit.2019.04.008
5	American Academy of Orthopaedic Surgeons	Management of Osteoarthritis of the Hip Evidence-Based Clinical Practice Guideline	N/A	2017	.	https://www.aaos.org/globalassets/quality-and-practice-resources/osteoarthritis-of-the-hip/oa-hip-cpg_6-11-19.pdf
6	Chen WH, Liu XX, Tong PJ, Zhan HS; Orthopaedic Professional Committee, Chinese Association of Research and Advancement of Chinese Traditional Medicine, China; Joint Professional Committee, Branch of Orthopaedic of Chinese Association of Integrative Medicine, China.	Diagnosis and management of knee osteoarthritis: Chinese medicine expert consensus (2015)	Chin J Integr Med	2016	26688182	10.1007/s11655-015-2432-7
7	Browne JA, Nho SJ, Goodman SB, Della Valle CJ.	American Association of Hip and Knee Surgeons, Hip Society, and Knee Society Position Statement on Biologics for Advanced Hip and Knee Arthritis	J Arthroplasty	2019	31005436	10.1016/j.arth.2019.03.068
8	Bruyere O, Cooper C, Pelletier JP, Maheu E,	A consensus statement on the European Society	Semin Arthritis	2016	26806188	10.1016/j.semarthrit.2015.11.

	Rannou F, Branco J, Luisa Brandi M, Kanis JA, Altman RD, Hochberg MC, Martel-Pelletier J, Reginster JY.	for Clinical and Economic Aspects of Osteoporosis and Osteoarthritis (ESCEO) algorithm for the management of knee osteoarthritis-From evidence-based medicine to the real-life setting	Rheum			010
9	Crossley KM, Stefanik JJ, Selfe J, Collins NJ, Davis IS, Powers CM, McConnell J, Vicenzino B, Bazett-Jones DM, Esculier JF, Morrissey D, Callaghan MJ.	2016 Patellofemoral pain consensus statement from the 4th International Patellofemoral Pain Research Retreat, Manchester. Part 1: Terminology, definitions, clinical examination, natural history, patellofemoral osteoarthritis and patient-reported outcome measures	Br J Sports Med	2016	27343241	10.1136/bjsports-2016-096384
10	Brosseau L, Wells GA, Pugh AG, Smith CA, Rahman P, ï¿½lvarez Gallardo IC, Toupin-April K, Loew L, De Angelis G, Cavallo S, Taki J, Marcotte R, Fransen M, Hernandez-Molina G, Kenny GP, Regnaux JP, Lefevre-Colau MM, Brooks S, Laferriere L, McLean L, Longchamp G.	Ottawa Panel evidence-based clinical practice guidelines for therapeutic exercise in the management of hip osteoarthritis	Clin Rehabil	2016	26400851	10.1177/0269215515606198
11	Brosseau L, Taki J, Desjardins B, Thevenot O, Fransen M, Wells GA, Imoto AM, Toupin-April K, Westby M, Gallardo ICï¿½, Gifford W, Laferriï¿½re L, Rahman P, Loew L, Angelis G, Cavallo S, Shallwani SM, Aburub A, Bennell KL, Van der Esch M, Simic M, McConnell S, Harmer A, Kenny GP, Paterson G, Regnaux JP, Lefevre-Colau MM, McLean L.	The Ottawa panel clinical practice guidelines for the management of knee osteoarthritis. Part one: introduction, and mind-body exercise programs	Clin Rehabil	2017	28183188	10.1177/0269215517691083
12	Brosseau L, Taki J, Desjardins B, Thevenot O, Fransen M, Wells GA, Mizusaki Imoto A, Toupin-April K, Westby M, ï¿½lvarez Gallardo IC, Gifford W, Laferriï¿½re L, Rahman P, Loew L, De Angelis G, Cavallo S, Shallwani SM, Aburub A, Bennell KL, Van der Esch M, Simic M, McConnell S, Harmer A, Kenny GP, Paterson G, Regnaux JP, Lefevre-Colau MM, McLean L.	The Ottawa panel clinical practice guidelines for the management of knee osteoarthritis. Part two: strengthening exercise programs	Clin Rehabil	2017	28183213	10.1177/0269215517691084
13	Brosseau L, Taki J, Desjardins B, Thevenot O, Fransen M, Wells GA, Mizusaki Imoto A, Toupin-April K, Westby M, ï¿½lvarez Gallardo IC, Gifford W, Laferriï¿½re L, Rahman P, Loew L, De Angelis G, Cavallo S, Shallwani SM, Aburub A, Bennell KL, Van der Esch M, Simic M, McConnell S, Harmer A, Kenny GP, Paterson G, Regnaux JP, Lefevre-Colau MM, McLean L.	The Ottawa panel clinical practice guidelines for the management of knee osteoarthritis. Part three: aerobic exercise programs	Clin Rehabil	2017	28183194	10.1177/0269215517691085
14	Brosseau L, Thevenot O, MacKiddie O, Taki J,	The Ottawa Panel guidelines on programmes	Clin Rehabil	2018	29911409	10.1177/0269215518780973

	Wells GA, Guitard P, Lï¿½onard G, Paquet N, Aydin SZ, Toupin-April K, Cavallo S, Moe RH, Shaikh K, Gifford W, Loew L, De Angelis G, Shallwani SM, Aburub AS, Mizusaki Imoto A, Rahman P, ï¿½lvarez Gallardo IC, Cosic MB, ï¿½sterï¿½s N, Lue S, Hamasaki T, Gaudreault N, Towheed TE, Koppikar S, Kjeken I, Mahendira D, Kenny GP, Paterson G, Westby M, Laferriï¿½re L, Longchamp G.	involving therapeutic exercise for the management of hand osteoarthritis				
15	Conrozier T, Monfort J, Chevalier X, Raman R, Richette P, Diraï¿½oglï¿½ D, Bard H, Baron D, Jerosch J, Migliore A, Henrotin Y.	EUROVISCO Recommendations for Optimizing the Clinical Results of Viscosupplementation in Osteoarthritis	Cartilage	2020	29926748	10.1177/1947603518783455
16	Arthroscopy Association of Canada, Wong I, Hiemstra L, Ayeni OR, Getgood A, Beavis C, Volesky M, Outerbridge R, Sheehan B, McCormack R, Litchfield R, Whelan D, Mohtadi N, Coady C, MacDonald PB.	Position Statement of the Arthroscopy Association of Canada (AAC) Concerning Arthroscopy of the Knee Joint-September 2017	Orthop J Sports Med	2018	29511702	10.1177/2325967118756597
17	Arthroscopy Association of Canada, Kopka M, Sheehan B, Degen R, Wong I, Hiemstra L, Ayeni O, Getgood A, Beavis C, Volesky M, Outerbridge R, Matache B.	Arthroscopy Association of Canada Position Statement on Intra-articular Injections for Knee Osteoarthritis	Orthop J Sports Med	2019	31367647	10.1177/2325967119860110
18	Ayhan FF, Sunar ?, Umay E, Kesk?n D, Altan L, D?nï¿½er F, Duruï¿½z T, Karalezl? N, Kuran B, Tuncer T.	The Turkish League Against Rheumatism Recommendations for the Management of Hand Osteoarthritis Under Guidance of the Current Literature and 2018 European League Against Rheumatism Recommendations	Arch Rheumatol	2020	33458653	10.46497/ArchRheumatol.2020.7693
19	Eymard F, Ornetti P, Maillet J, Noel ï¿½, Adam P, Legrï¿½-Boyer V, Boyer T, Allali F, Gremeaux V, Kaux JF, Louati K, Lamontagne M, Michel F, Richette P, Bard H; GRIP (Groupe de Recherche sur les Injections de PRP, PRP Injection Research Group).	Intra-articular injections of platelet-rich plasma in symptomatic knee osteoarthritis: a consensus statement from French-speaking experts	Knee Surg Sports Traumatol Arthrosc	2020	32583023	10.1007/s00167-020-06102-5
20	Hawk C, Whalen W, Farabaugh RJ, Daniels CJ, Minkalis AL, Taylor DN, Anderson D, Anderson K, Crivelli LS, Cark M, Barlow E, Paris D, Sarnat R, Weeks J.	Best Practices for Chiropractic Management of Patients with Chronic Musculoskeletal Pain: A Clinical Practice Guideline	J Altern Complement Med	2020	32749874	10.1089/acm.2020.0181
21	Geenen R, Overman CL, Christensen R, ï¿½senlï¿½f P, Capela S, Huisinga KL, Husebï¿½ MEP, Kï¿½ke AJA, Paskins Z, Pitsillidou IA, Savel C, Austin J, Hassett AL, Severijns G, Stoffer-Marx M, Vlaeyen JWS, Fernï¿½ndez-de-Las-Peï¿½as C, Ryan SJ,	EULAR recommendations for the health professional's approach to pain management in inflammatory arthritis and osteoarthritis	Ann Rheum Dis	2018	29724726	10.1136/annrheumdis-2017-212662

	Bergman S.					
22	Department of Veterans Affairs	VA/DoD CLINICAL PRACTICE GUIDELINE FOR THE NON-SURGICAL MANAGEMENT OF HIP & KNEE OSTEOARTHRITIS	N/A	2020	.	https://www.healthquality.va.gov/guidelines/CD/OA/VADoDOACPG.pdf
23	Iolascon G, Ruggiero C, Fiore P, Mauro GL, Moretti B, Tarantino U.	Multidisciplinary integrated approach for older adults with symptomatic osteoarthritis: SIMFER and SI-GUIDA Joint Position Statement	Eur J Phys Rehabil Med	2020	31742367	10.23736/S1973-9087.19.05837-4
24	Kloppenburg M, Kroon FP, Blanco FJ, Doherty M, Dziedzic KS, Greibrokk E, Haugen IK, Herrero-Beaumont G, Jonsson H, Kjeken I, Maheu E, Ramonda R, Ritt MJ, Smeets W, Smolen JS, Stamm TA, Szekanecz Z, Wittoek R, Carmona L.	2018 update of the EULAR recommendations for the management of hand osteoarthritis	Ann Rheum Dis	2019	30154087	10.1136/annrheumdis-2018-213826
25	Manchikanti L, Centeno CJ, Atluri S, Albers SL, Shapiro S, Malanga GA, Abd-Elsayed A, Jerome M, Hirsch JA, Kaye AD, Aydin SM, Beall D, Buford D, Borg-Stein J, Buenaventura RM, Cabaret JA, Calodney AK, Candido KD, Cartier C, Latchaw R, Diwan S, Dodson E, Fausel Z, Fredericson M, Gharibo CG, Gupta M, Kaye AM, Knezevic NN, Kosanovic R, Lucas M, Manchikanti MV, Mason RA, Mautner K, Murala S, Navani A, Pampati V, Pastoriza S, Pasupuleti R, Philip C, Sanapati MR, Sand T, Shah RV, Soin A, Stemper I, Wargo BW, Hernigou P.	Bone Marrow Concentrate (BMC) Therapy in Musculoskeletal Disorders: Evidence-Based Policy Position Statement of American Society of Interventional Pain Physicians (ASIPP)	Pain Physician	2020	32214287	missing5
26	Polido-Pereira J, Serra S, Teixeira F, Ponte C, Cerqueira M, Cruz M, Araï¿½jo F, Barros R, Costa T, Santos-Faria D, Lopes C, Madruga-Dias J, Oliveira M, Teixeira R, Vilar A, Falcï¿½o S, Saraiva F, Figueiredo G.	Portuguese recommendations for the use of ultrasound in rheumatology	Acta Reumatol Port	2019	31249273	missing7
27	Rausch Osthoff AK, Niedermann K, Braun J, Adams J, Brodin N, Dagfinrud H, Duruoz T, Esbensen BA, Gï¿½nther KP, Hurkmans E, Juhl CB, Kennedy N, Kiltz U, Knittle K, Nurmohamed M, Pais S, Severijns G, Swinnen TW, Pitsillidou IA, Warburton L, Yankov Z, Vliet Vlieland TPM.	2018 EULAR recommendations for physical activity in people with inflammatory arthritis and osteoarthritis	Ann Rheum Dis	2018	29997112	10.1136/annrheumdis-2018-213585
28	Rillo O, Riera H, Acosta C, Liendo V, Bolaï¿½os J, Monterola L, Nieto E, Arape R, Franco LM, Vera M, Papasidero S, Espinosa R, Esquivel JA, Souto R, Rossi C, Molina JF, Salas J, Ballesteros F, Radrigan F, Guibert M, Reyes G, Chico A, Camacho W, Urioste L, Garcia A, Iraheta I, Gutierrez CE, Aragï¿½n R, Duarte M, Gonzalez	PANLAR Consensus Recommendations for the Management in Osteoarthritis of Hand, Hip, and Knee	J Clin Rheumatol	2016	27660931	10.1097/RHU.0000000000000449

	M, Castaï¿½eda O, Angulo J, Coimbra I, Munoz-Louis R, Saenz R, Vallejo C, Briceï¿½o J, Acuï¿½a RP, De Leï¿½n A, Reginato AM, Mï¿½ller I, Caballero CV, Quintero M.					
29	Sakellariou G, Conaghan PG, Zhang W, Bijlsma JWJ, Boyesen P, D'Agostino MA, Doherty M, Fodor D, Kloppenburg M, Miese F, Naredo E, Porcheret M, Iagnocco A.	EULAR recommendations for the use of imaging in the clinical management of peripheral joint osteoarthritis	Ann Rheum Dis	2017	28389554	10.1136/annrheumdis-2016-210815
30	Sellam J, Courties A, Eymard F, Ferrero S, Latourte A, Ornetti P, Bannwarth B, Baumann L, Berenbaum F, Chevalier X, Ea HK, Fabre MC, Forestier R, Grange L, Lellouche H, Maillet J, Mainard D, Perrot S, Rannou F, Rat AC, Roux CH, Senbel E, Richette P; French Society of Rheumatology.	Recommendations of the French Society of Rheumatology on pharmacological treatment of knee osteoarthritis	Joint Bone Spine	2020	32931933	10.1016/j.jbspin.2020.09.004
31	Tuncer T, Cay FH, Altan L, Gurer G, Kacar C, Ozcakir S, Atik S, Ayhan F, Durmaz B, Eskiyurt N, Genc H, GokceKutsal Y, Gunaydin R, Hepguler S, Hizmetli S, Kaya T, Kurtais Y, Saridogan M, Sindel D, Sutbeyaz S, Sendur OF, Ugurlu H, Unlu Z.	2017 update of the Turkish League Against Rheumatism (TLAR) evidence-based recommendations for the management of knee osteoarthritis	Rheumatol Int	2018	29777340	10.1007/s00296-018-4044-y
32	Brazilian Medical Association, Silvinato A, Bernardo WM.	Inflammatory arthritis or osteoarthritis of the knee - Efficacy of intra-joint infiltration of methylprednisolone acetate versus triamcinolone acetonide or triamcinolone hexacetonide	Rev Assoc Med Bras (1992)	2017	29267483	10.1590/1806-9282.63.10.827
33	Cibulka MT, Bloom NJ, Enseki KR, Macdonald CW, Woehrle J, McDonough CM.	Hip Pain and Mobility Deficits-Hip Osteoarthritis: Revision 2017	J Orthop Sports Phys Ther	2017	28566053	10.2519/jospt.2017.0301
34	Khazzam M, Gee AO, Pearl M.	Management of Glenohumeral Joint Osteoarthritis	J Am Acad Orthop Surg	2020	32986386	10.5435/JAAOS-D-20-00404
35	Zhang Z, Huang C, Jiang Q, Zheng Y, Liu Y, Liu S, Chen Y, Mei Y, Ding C, Chen M, Gu X, Xing D, Gao M, He L, Ye Z, Wu L, Xu J, Yang P, Zhang X, Zhang Y, Chen J, Lin J, Zhao L, Li M, Yang W, Zhou Y, Jiang Q, Chu CQ, Chen Y, Zhang W, Tsai WC, Lei G, He D, Liu W, Fang Y, Wu D, Lin J, Wei CC, Lin HY, Zeng X.	Guidelines for the diagnosis and treatment of osteoarthritis in China (2019 edition)	Ann Transl Med	2020	33178745	10.21037/atm-20-4665
36	Royal Australian College of General Practitioners (RACGP)	Guideline for the management of knee and hip osteoarthritis; Second Edition	N/A	2018	.	https://www.racgp.org.au/getattachment/71ab5b77-afdf-4b01-90c3-04f61a910be6/Guideline-for-the-management-of-knee-and-hip-osteoarthritis.aspx

37	Siemieniuk RAC, Harris IA, Agoritsas T, Poolman RW, Brignardello-Petersen R, Van de Velde S, Buchbinder R, Englund M, Lytvyn L, Quinlan C, Helsingen L, Knutsen G, Olsen NR, Macdonald H, Hailey L, Wilson HM, Lydiatt A, Kristiansen A.	Arthroscopic surgery for degenerative knee arthritis and meniscal tears: a clinical practice guideline	BMJ	2017	28490431	10.1136/bmj.j1982
38	van Doormaal MCM, Meerhoff GA, Vliet Vlieland TPM, Peter WF	A clinical practice guideline for physical therapy in patients with hip or knee osteoarthritis	Musculoskeletal Care	2020	32643252	10.1002/msc.1492
39	Petzke F, Bock F, HÃ¼ppe M, Nothacker M, Norda H, Radbruch L, Schiltenwolf M, Schuler M, TÃ¶lle T, Viniol A, HÃ¤user W	Long-term opioid therapy for chronic noncancer pain: second update of the German guidelines	Pain Rep	2020	32904018	10.1097/PR9.0000000000000840
40	National Institute for Health and Care Excellence (NICE)	Osteoarthritis: care and management	N/A	2020	.	https://www.nice.org.uk/guidance/cg177/resources/osteoarthritis-care-and-management-pdf-35109757272517
41	Ruiz IbÃ¡n MA, MaculÃ© F, Torner P, Gil Garay E, Oteo-Ãlvaro A, LÃ³pez MillÃ¡n JM, DÃ-az Heredia J, Loza E.	SECOT-GEDOS consensus on pre-surgical pain management in knee and hip arthrosis	Rev Esp Cir Ortop Traumatol	2015	25435293	10.1016/j.recot.2014.09.005
